# Fbrsl1 is required for cranial neural crest development and reflects a conserved function of the human disease-associated protein

**DOI:** 10.1242/dmm.052472

**Published:** 2025-11-24

**Authors:** Sarah Gerstner, Hanna Berger-Santangelo, Gina Kastens, Tamara Scholtes, Stella Wäschenbach, Silke Pauli, Annette Borchers

**Affiliations:** ^1^ Department of Biology, Molecular Embryology, Philipps-University Marburg, 35043 Marburg, Germany; ^2^ Institute of Human Genetics, University Medical Center Göttingen, Heinrich-Düker-Weg 12, 37073 Göttingen, Germany

**Keywords:** Craniofacial malformation, Neural crest development, FBRSL1, *Xenopus*, Splicing, P53

## Abstract

We recently identified a rare complex syndrome with craniofacial malformations caused by truncating variants in fibrosin-like 1 (*FBRSL1*). To investigate the function of Fbrsl1 in craniofacial development, we used the *Xenopus laevis* model to study the cranial neural crest (NC). While Fbrsl1 was largely dispensable for NC induction and early migration, its loss of function impaired NC differentiation and cartilage formation. This was accompanied by increased expression of p53 and cleaved caspase-3, as well as by exon skipping in the *mdm2* gene, a negative regulator of p53. Fbrsl1 may directly affect splicing of *mdm2*, as we find that FBRSL1 interacts with the splicing factor SF3B1. Notably, pharmacological inhibition of p53 partially rescued the craniofacial phenotype, suggesting that p53-mediated apoptosis underlies the NC defects caused by loss of Fbrsl1 function.

## Teaser

**Summary:** FBRSL1 interacts with the splicing factor SF3B1 and is required for survival of *Xenopus* cranial neural crest cells.

## INTRODUCTION

In 2020, we first identified heterozygous truncating variants in *FBRSL1*, a member of the AUTS2 gene family, as the cause of a previously unrecognized syndrome (Ufartes et al., 2020). Patients presented with global developmental delay, respiratory distress and feeding difficulties in the neonatal period, craniofacial malformations, lack of active speech, postnatal microcephaly, growth retardation, contractures and a varying degree of internal organ malformations, such as heart defects (Bukvic et al., 2024; Pauli et al., 2021; Ufartes et al., 2020). Craniofacial malformations range in severity from dysmorphic features – including micro- and retrognathia, epicanthal folds, low set ears and deep and/or broad nasal bridges – to more pronounced anomalies such as cleft palate; hearing impairment was also observed (Bukvic et al., 2024; Pauli et al., 2021). To date, four patients have been described, and the identified variants all affect a very narrow region of the FBRSL1 protein (Bukvic et al., 2024; Ufartes et al., 2020). FBRSL1 undergoes differential splicing, and we found one long isoform (I1, NM_001142641.2), as well as two short N-terminal isoforms that contain an alternative exon 3 introducing a premature stop codon (I3.1, NM_001382741.1 and I3.2, NM_001382742.1). While the long isoform I1 contains a AUTS2 domain similar to its paralog AUTS2, both short isoforms lack this conserved region (Ufartes et al., 2020). All pathogenic variants cluster either in exon 2 or in the alternative exon 3 of FBRSL1. Two patients carry nonsense variants (c.487C>T, p.Q163* and c.332G>A, p.W111*), while the other two harbor frameshift variants that introduce a premature stop codon (c.581-603del and c.371dupC) (Bukvic et al., 2024; Ufartes et al., 2020). These findings suggest that truncation of the N-terminal domain of FBRSL1 may represent a key pathogenic mechanism and highlight the need to analyze the function of FBRSL1 in embryonic development.

At the time of the discovery of the FBRSL1-associated syndrome, the function of FBRSL1 was largely unknown and, in particular, its role in embryonic development was unclear. Using the *Xenopus laevis* model system, we found that morpholino-mediated knockdown of Fbrsl1 caused severe craniofacial defects, reduced brain size, cranial and motor neuron outgrowth defects, and severely hypoplastic hearts (Berger et al., 2024; Ufartes et al., 2020). Concerning heart development, we recently showed that Fbrsl1 is required for the development of the first heart field, which contributes to the ventricle and the atria, but not for the development of the second heart field, which gives rise to the outflow tract (Berger et al., 2024). The heart and craniofacial defects of *fbrsl1* morphants could be rescued by the short human N-terminal isoform I3.1, but not by the long isoform I1, which carries the AUTS2 domain. N-terminal isoforms carrying the patient variants were also not able to rescue the morphant phenotype, suggesting that the N-terminal isoform is crucial for embryonic development (Berger et al., 2024; Ufartes et al., 2020).

Although all four patients carrying truncating *FBRSL1* variants exhibit craniofacial malformations of varying severity (Pauli et al., 2021), the precise role of *FBRSL1* in craniofacial development remains unclear. Given that craniofacial development is closely linked to the biology of NC cells (Roth et al., 2021; Siismets and Hatch, 2020), FBRSL1 may play a role in NC cell function or regulation. NC cells are a transient, highly migratory embryonic cell population that arises at the neural plate border. They undergo an epithelial-to-mesenchymal transition and migrate along predetermined routes to specific locations in the embryo, where they give rise to a wide range of derivatives (Szabo and Mayor, 2018). The cranial NC is essential for craniofacial development, as it gives rise to cartilage, bone, connective and nervous tissue of the face and part of the skull (Bronner and LeDouarin, 2012; Dash and Trainor, 2020). *Xenopus fbrsl1* morphant embryos show craniofacial defects, and collagen II staining confirmed cartilage hypoplasia with a severe reduction in Meckel's cartilage, the ceratohyal cartilage and the branchial arches (Ufartes et al., 2020). To investigate whether defects in NC development underlie the craniofacial defects seen in patients, we used here the *Xenopus laevis* model to systematically examine the effects of Fbrsl1 loss of function on NC development.

## RESULTS

### Fbrsl1 is required for later stages of NC development, but not for NC induction and specification

Patients affected by the FBRSL1-associated syndrome show craniofacial malformations (Bukvic et al., 2024; Ufartes et al., 2020), which are an indication of defects in NC development. Previously, we observed that morpholino-mediated knockdown of Fbrsl1 in *Xenopus laevis* leads to craniofacial defects, including a severe reduction in craniofacial cartilage (Ufartes et al., 2020). Therefore, we turned here to the *Xenopus* system to further dissect at which steps of NC development Fbrsl1 might be required.

**Fig. 1.**
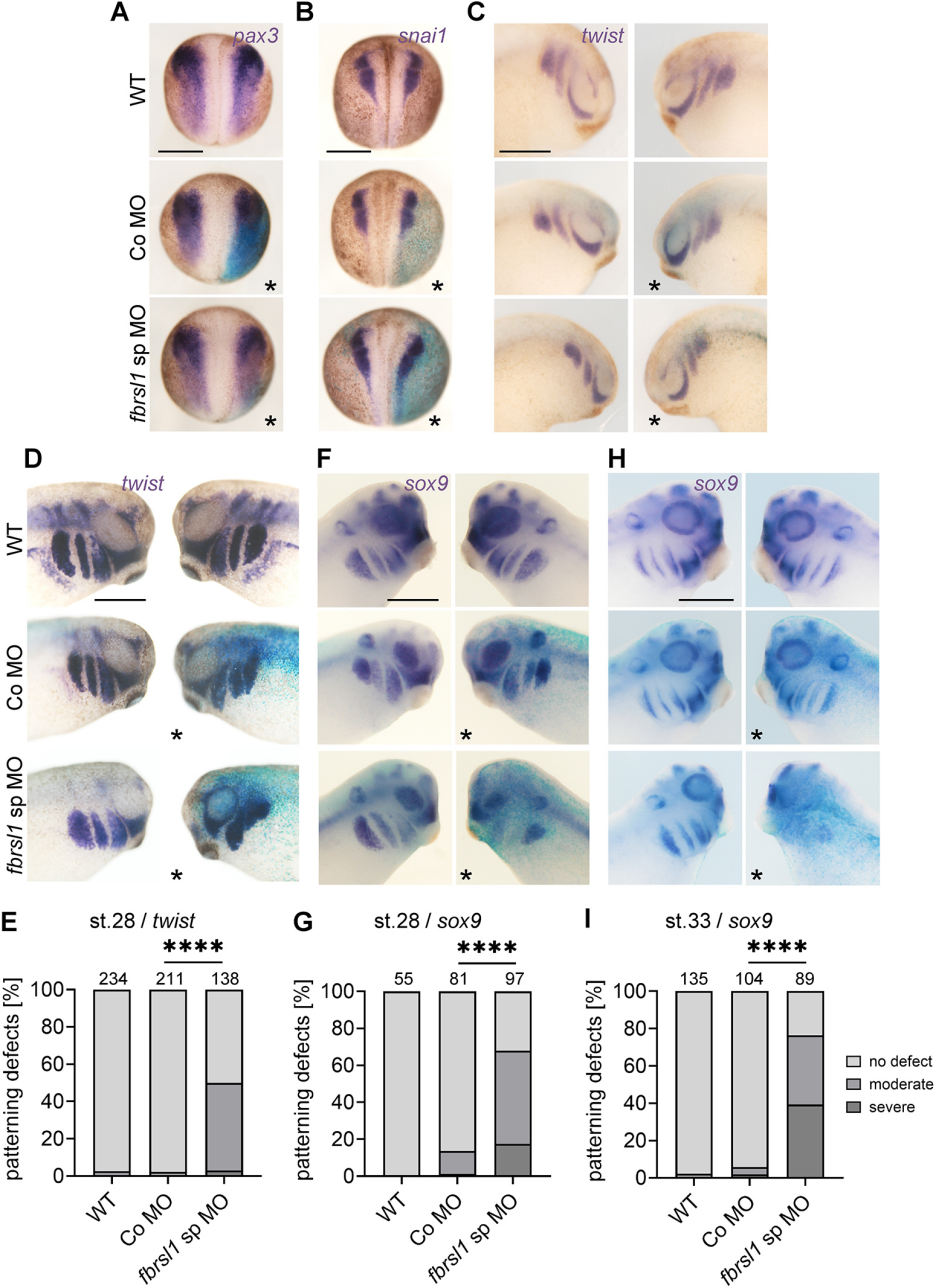
**Fbrsl1 loss of function does not affect NC induction, specification and early migration, but later stages of NC development.** Embryos were uninjected, injected with 15 ng Co MO or injected with 10 ng *fbrsl1* sp MO and 80-100 pg *lacZ* mRNA at the two-cell stage and analyzed by whole-mount *in situ* hybridization using NC markers as indicated. Asterisks indicate the injected side, which is also visible by the blue LacZ staining. WT, wild type (uninjected control). Scale bars: 500 µm. (A) Dorsal views of a stage 14 embryos analyzed for expression of the neural plate border marker *pax3*. (B) Dorsal views of stage 16 embryos analyzed for the expression of *snai1*, indicating premigratory NC cells. (C) Lateral views of stage 23 embryos showing early migrating NC cells marked by *twist* expression. (D) Lateral views of stage 28 embryos showing patterning defects of *twist-*expressing NC cells compared to controls. (F) The same was seen for the *sox9* expression pattern at stage 28. (H) These defects were even more severe when embryos were analyzed at stage 33. (E,G,I) Data relating to D,F,H, respectively, presented as a percentage of the total, based on at least three independent experiments. The number of analyzed embryos is indicated; ****P≤0.0001 (two-sided Fisher's exact test).

*Xenopus laevis* embryos were injected with a control morpholino (Co MO) or a splice blocking *fbrsl1* MO (*fbrsl1* sp MO) in combination with *lacZ* RNA for lineage tracing in one blastomere at the two-cell stage. Embryos were cultured to different stages and then analyzed by *in situ* hybridization (Fig. 1). We started our analysis at neurula stages and found that only a few *fbrsl1* MO-injected embryos showed defects in the expression of the neural border marker *pax3* or the NC specification marker *snai1* (Fig. 1A,B). The vast majority showed no differences on the *fbrsl1* MO-injected side compared to the uninjected side or to non-injected control embryos (Fig. S1A,B). To assess early positioning of NC cells, *twist* (*twist1*) expression was analyzed at stage 23. Despite a small but statistically significant increase in defects, most embryos developed normally (Fig. 1C, Figs S1C and S2A). Comparable results were obtained using a translation-blocking *fbrsl1* MO (Fig. S1A-C)*.* Analyzing the ratio of the *twist* expression area between the injected side and the uninjected side of randomly selected embryos did not reveal significant defects (Fig. S3A). These data suggest that Fbrsl1 does not play a major role in NC induction or early NC positioning.

We continued to analyze whether Fbrsl1 functions at later stages of NC development by analyzing the expression pattern of the NC marker *twist* and *sox9* at tailbud stage 28 (Fig. 1D-G). Notably the *fbrsl1* sp MO-injected embryos showed defects on the injected side (Fig. 1D-G). These defects ranged from NC cells that showed abnormal positioning to a severe reduction in *twist* or *sox9* expression on the injected side (Fig. S2B,C). In addition, quantification of the *twist* or *sox9* expression areas, including calculation of the ratio between injected and uninjected sides, demonstrated a significant decrease in expression on the injected side (Fig. S3B,C). Similar defects were also observed when the translation-blocking *fbrsl1* MO (*fbrsl1* tb MO) was injected (Fig. S1D,E). These defects were even more evident at late tailbud stage 33, where we observed a severe reduction in *sox9*-expressing cells on the injected side (Fig. 1H,I, Figs S1F, S2D and S3D). Thus, consistent with a defect in craniofacial and cartilage development in *fbrsl1* morphants, we observe here defects in NC cell development at tailbud stages, which seem to progressively worsen as the embryos continue to develop.

### The short human N-terminal isoform of FBRLS1 rescues NC defects of *fbrsl1* tailbud stage morphants

**Fig. 2.**
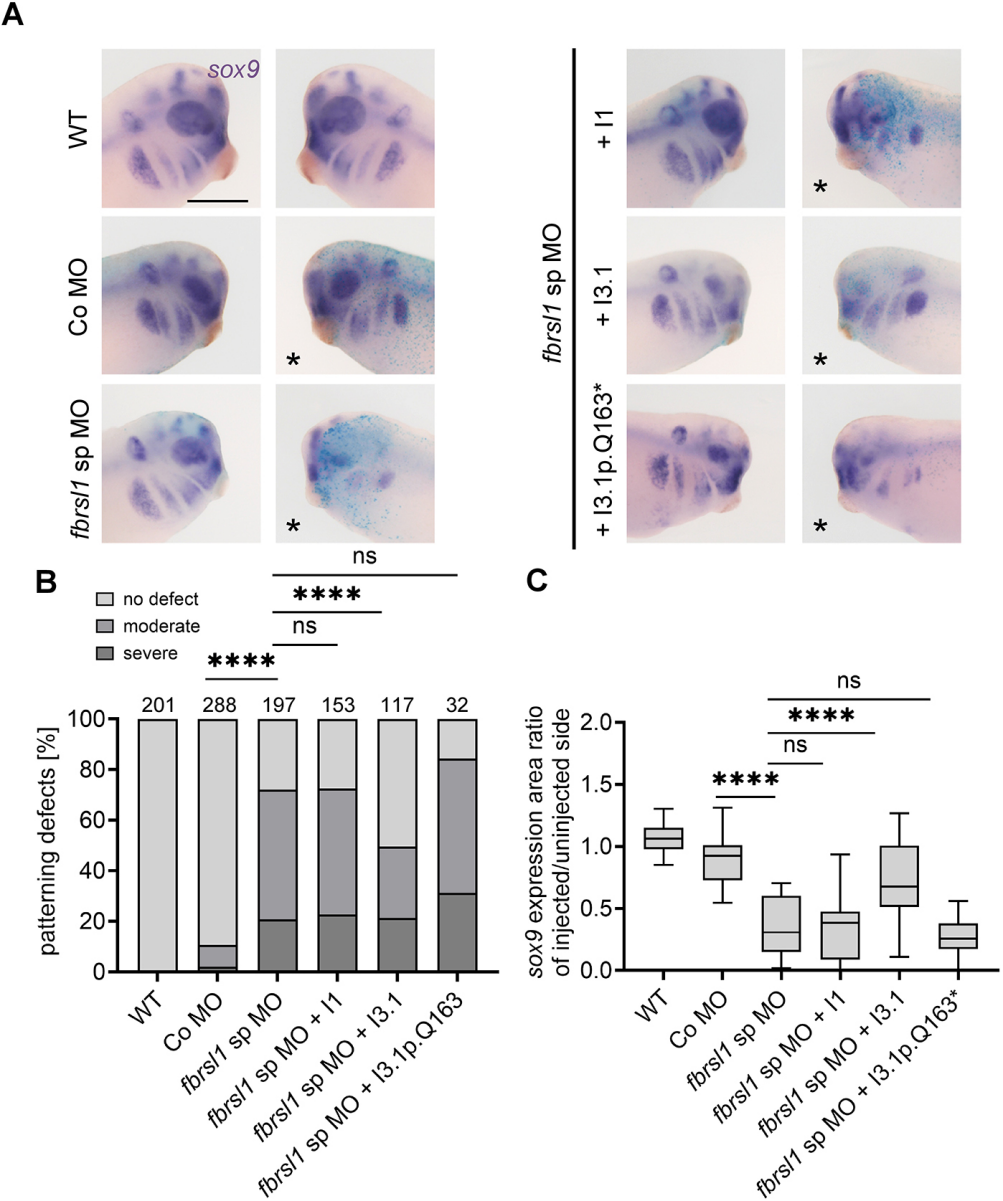
**NC patterning defects are partially rescued by the human short N-terminal FBRSL1 isoform.** (A) Embryos were injected at the two-cell stage with 10 ng of the respective MO alone or in combination with 200 pg of the respective plasmids. 80 pg *LacZ* RNA was co-injected as a lineage tracer. Embryos were analyzed at stage 28 by *sox9* whole-mount *in situ* hybridization. Embryos are shown from the lateral side. Scale bar: 500 µm. Injection of *fbrsl1* sp MO caused a significant reduction of *sox9* staining on the injected side compared to controls. Co-injection of the short human *FBRSL1* isoform I3.1 was able to significantly restore this knockdown phenotype, whereas co-injection of the long isoform I1 or the short isoform I3.1p.Q163*, carrying a patient variant, were not. (B) Data in the graph are presented as a percentage of the total, based on at least three independent experiments. The numbers of analyzed embryos are indicated; ns, not significant. ****P≤0.0001 (two-sided Fisher's exact test). (C) Graph presenting the ratio of the *sox9* expression area between the injected and the uninjected side of 20 randomly selected embryos per condition; ns, not significant. ****P≤0.0001 (one-way ANOVA with Tukey's multiple comparison test). Boxplots indicate the median (horizontal line), the boxes extend from the 25th to the 75th percentile, with Tukey's whiskers set at 1.5 times the IQR.

To confirm that the observed defects in NC cell development of *fbrsl1* morphants are specific for Fbrsl1 loss of function, we performed rescue experiments using human FBRSL1 constructs. We used the long isoform (I1), the short N-terminal isoform (I3.1) lacking the AUTS2 domain and containing an alternative exon 3 (Ufartes et al., 2020), and the short N-terminal isoform carrying the pathogenic patient variant, p.Q163*, for our rescue experiments. Plasmids were injected to limit protein expression to stages after the mid-blastula transition and to avoid potential effects during early developmental stages. Embryos were injected in one blastomere at the two-cell stage with MO and *lacZ* RNA alone or in combination with different FBRSL1 plasmids. At tailbud stages, NC migration was analyzed by *sox9 in situ* hybridization. As expected, loss of Fbrsl1 function resulted in defects in the *sox9* expression pattern compared to controls (Fig. 2). These defects were partially rescued by co-injection of the short N-terminal FBRSL1 isoform I3.1, but not by the long isoform I1. The short N-terminal isoform carrying the pathogenic patient variant I3.1p.Q163* was also not able to rescue loss of Fbrsl1 function (Fig. 2A,B). This result was confirmed by quantification of the *sox9* expression area of the injected versus the uninjected side (Fig. 2C). Thus, these data suggest that the human N-terminal isoform of FBRSL1 is crucial for late NC development and that the patient variants likely compromise this function.

### Fbrsl1 does not control NC cell morphology or migration behavior

**Fig. 3.**
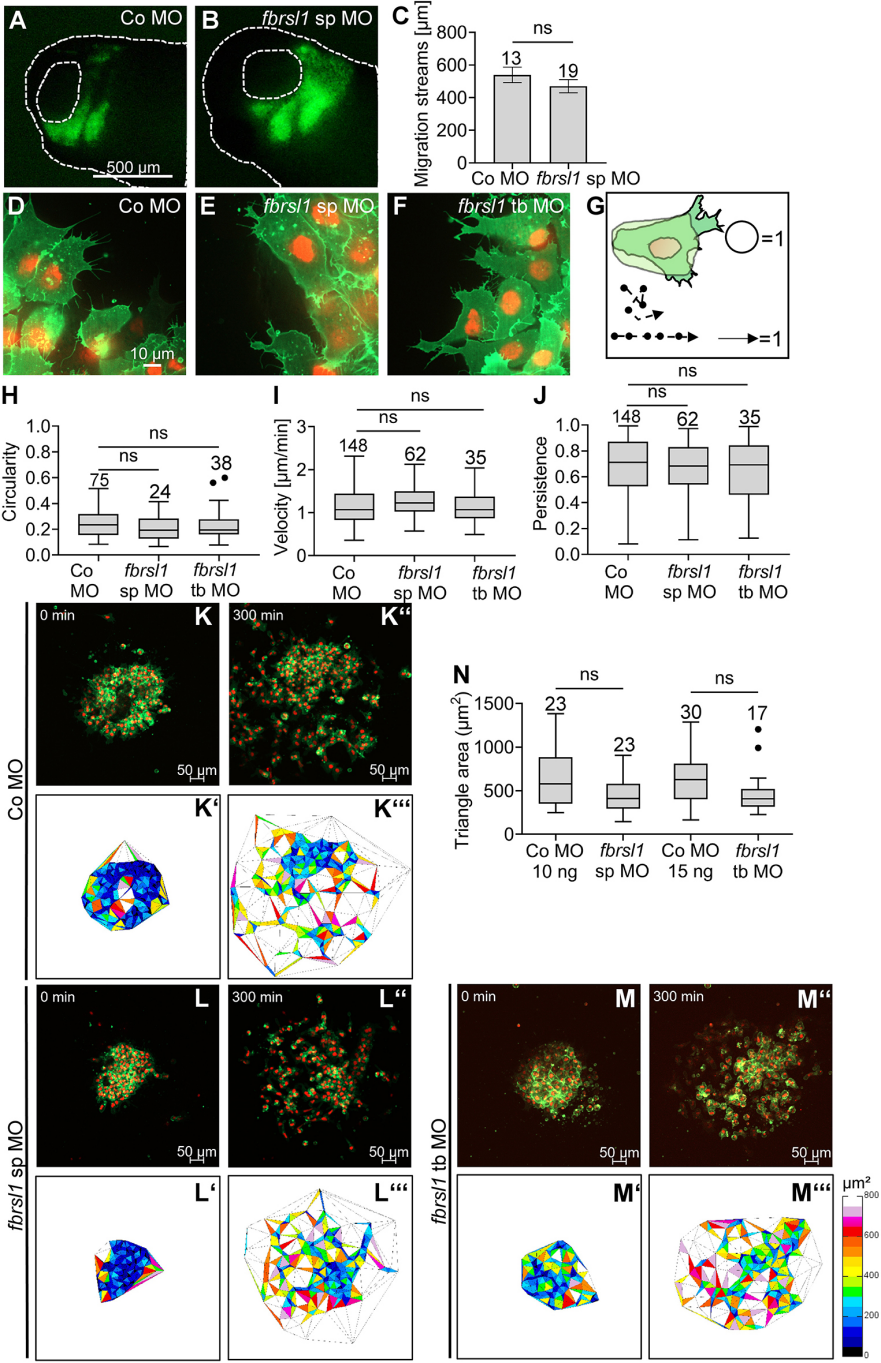
**Fbrsl1 loss of function does not affect the migration of NC cells.** (A,B) Transplantation assay. Embryos were injected with 10 ng Co MO or 10 ng *fbrsl1* sp MO in combination with either 150 pg *mbGFP* or *mRFP* mRNA. Fluorescently labeled NC cells were transplanted into wild-type embryos at stage 18 and NC cell migration was analyzed at stage 28. Fbrsl1-depleted NC cells showed no significant difference in migration compared to control cells. (C) Data from the experiments in A and B showing the average length of migration streams of the transplanted NC cells. Data are mean±s.e.m.; the number of analyzed transplants are indicated; ns, not significant (two-tailed unpaired Student's *t*-test). (D-N) Explant assay results. Embryos were injected with 10-15 ng Co MO (D,K-K‴), with 10 ng *fbrsl1* sp MO (E,L-L‴) or with 15 ng *fbrsl1* tb MO (F,M-M‴) in combination with 150 pg *mbGFP* RNA and 250 pg *H2B mCherry* mRNA. (D-F) NC cells were explanted at stage 18, cultivated for 2-3 h and imaged using spinning disk microscopy. (D) Co MO-injected explants form protrusions. (E,F) *fbrsl1* morphant explants form protrusions. (G) Cell circularity and persistence of migration; a complete circle has a circularity of 1, cells migrating in a straight line have a persistence of 1. (H-J) Graphs analyzing cell shape (circularity) (H), speed (velocity) (I) or directionality (persistence) (J) revealing no difference in NC morphology and migration behavior. ns, not significant [Kruskal–Wallis test; boxplots indicate the median (horizontal line), the boxes extend from the 25th to the 75th percentile, with Tukey's whiskers set at 1.5 times the IQR, number of evaluated cells are indicated; dots represent outliers]. (K-M‴) NC cells were imaged for 5 h and NC dispersion was analyzed using Delaunay triangulation. Explants are shown at the start of the experiment (K-M) and after 5 h (K″-M″). The respective triangles between migrating cells are indicated in K′-M′ and K‴-M‴. Fbrsl1 loss of function did not lead to significant defects compared to control cells. (N) Boxplot showing mean triangle area per explant. ns, not significant (Kruskal–Wallis test). Boxplots indicate the median (horizontal line), the boxes extend from the 25th to the 75th percentile, with Tukey‘s whiskers set at 1.5 times the IQR. Numbers of evaluated explants are indicated; dots represent outliers.

Next, we aimed to dissect which processes might be affected by Fbrsl1 loss of function at tailbud stages. During tailbud stages, NC cells are migrating into the branchial arches where they later differentiate into distinct derivatives; therefore, migration or the genetic network relevant for craniofacial differentiation could be affected in *fbrsl1* morphants. First, we focused on NC migration. To analyze if Fbrsl1 has a cell-autonomous function in NC cells, we performed transplantation experiments (Fig. 3A-C). Embryos were injected with MOs in combination with *GFP* or *RFP* RNA in one blastomere at the two-cell stage. At early neurula stages, premigratory fluorescent NC cells were grafted into wild-type embryos from which the endogenous NC cells had been removed. The embryos were cultured until tailbud stages, when the migration of the transplanted NC cells was analyzed. Interestingly, both control and *fbrsl1* morphant NC cells showed the typical cranial NC migration pattern, with distinct streams of migrating cells clearly visible (Fig. 3A,B). Furthermore, the length of the migrating NC streams was similar in *fbrsl1* morphant NC cells compared to controls (Fig. 3C).

Next, we took a closer look at the cellular level of NC migration. Therefore, embryos were injected with MOs in combination with *mbGFP* RNA and *H2B mCherry* mRNA to label the cell membrane and the nucleus, respectively. NC cells were explanted at premigratory stages and cultured on a fibronectin matrix for 2-3 h, then imaged using spinning disk microscopy. Independent of the injected constructs, all explanted NC cells formed protrusions (Fig. 3D-F). Consistently, analysis of the cell shape revealed no significant defects in the circularity of the NC cells (Fig. 3G,H). Furthermore, manual tracking of the cells demonstrated that there was no change in the velocity (Fig. 3I) or the persistence of migration in *fbrsl1* morphant NC cells compared to controls (Fig. 3G,J). In addition, NC dispersion was determined by Delaunay triangulation followed by calculation of the mean triangle size in explanted NC cells, which were cultured for 5 h (Fig. 3K-N). Although there was a slight decrease in mean triangle size in *fbrsl1* morphant NC cells compared to controls, we did not observe any significant defects (Fig. 3N). Thus, these data indicate that Fbrsl1 is not required for NC migration.

### Fbrsl1 is required for the expression of transcription factors relevant for cartilage development

**Fig. 4.**
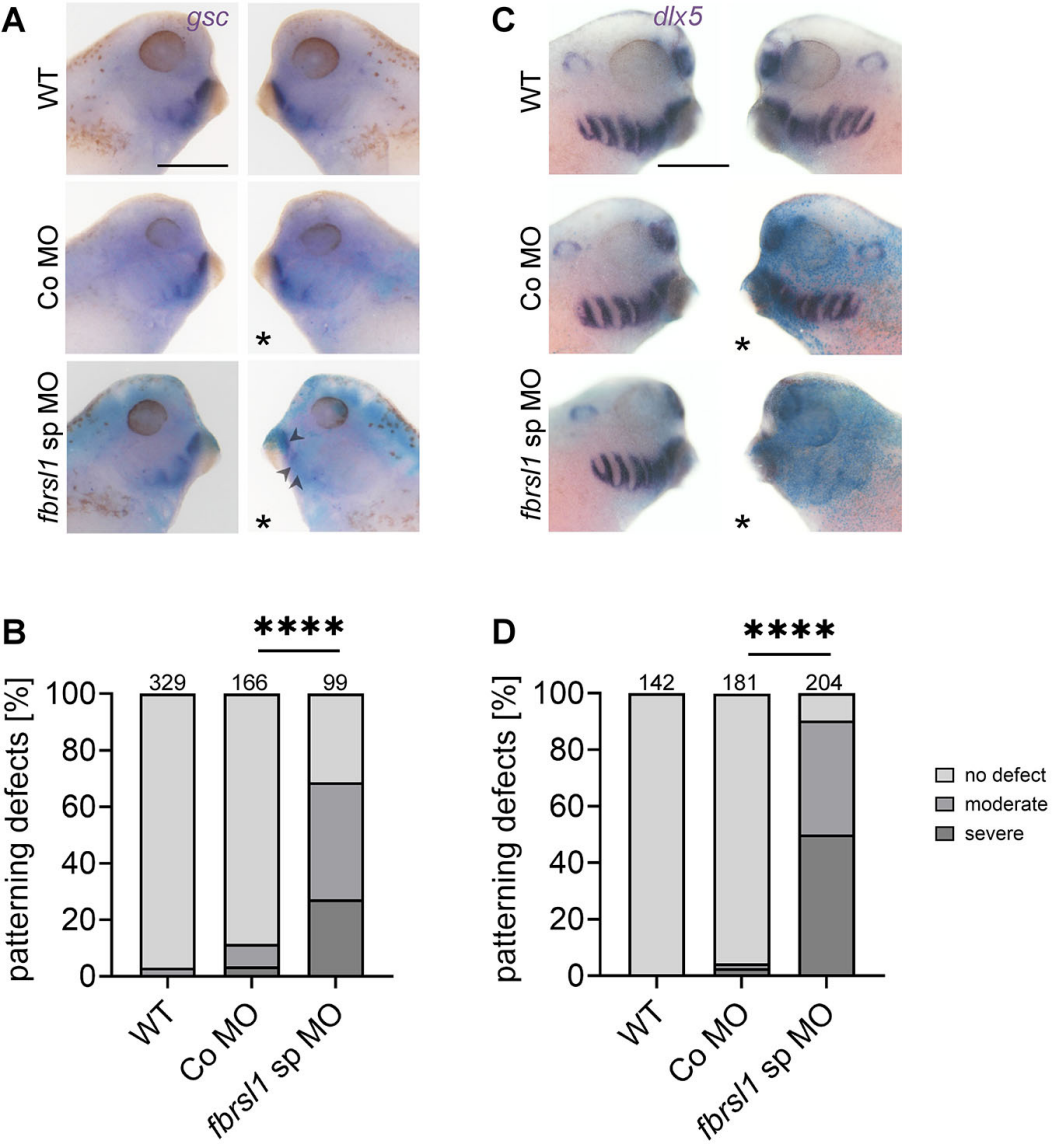
**Fbrsl1 is required for *gsc* and *dlx* expression.** (A,C) Embryos were injected at the two-cell stage with 10 ng of Co MO or *fbrsl1* sp MO, together with 80-100 pg of *lacZ* mRNA for lineage tracing. At stage 33-35, embryos were analyzed by whole-mount *in situ* hybridization for *gsc* or *dlx5* expression. Lateral views of the embryos are shown. Asterisks indicate the injected side. Scale bar: 500 µm. (A) *fbrsl1* morphants show a reduction in *gsc* expression compared to controls. Arrowheads mark the affected area. (B) Graph summarizing defects in the *gsc* expression pattern of three independent experiments. (C) *dlx5* expression is reduced in *fbrsl1* morphant embryos. (D) Graph summarizing defects in the *dlx5* expression pattern of four independent experiments. Data in B and D are presented as a percentage of the total. The number of analyzed embryos are indicated; ****P≤0.0001 (two-sided Fisher's exact test). *fbrsl1* sp MO images in A and C are also shown in Fig. S2.

As we did not observe any defects in the migration of cranial NC cells of *fbrsl1* morphant embryo, we analyzed if later steps of the developmental program required for skeletal morphogenesis are affected by Fbrsl1 loss of function. To this end, embryos were injected with MOs in combination with the lineage tracer *lacZ* in one blastomere at the two-cell stage. At late tailbud stages, the expression of transcription factors relevant for cartilage development, such as *gsc* and *dlx5*, was analyzed by *in situ* hybridization. *gsc* is expressed in the ventral pharyngeal arches, while *dlx5* is expressed in intermediate and ventral pharyngeal arches (Square et al., 2015). The expression of *gsc* (Fig. 4A,B, Fig. S2E) as well as *dlx5* (Fig. 4C,D, Fig. S2F) was significantly reduced in the pharyngeal arches. These data show that Fbrsl1 loss of function causes severe defects in the expression of factors relevant for cartilage development.

### Fbrsl1 depletion causes p53-mediated apoptosis of NC cells

**Fig. 5.**
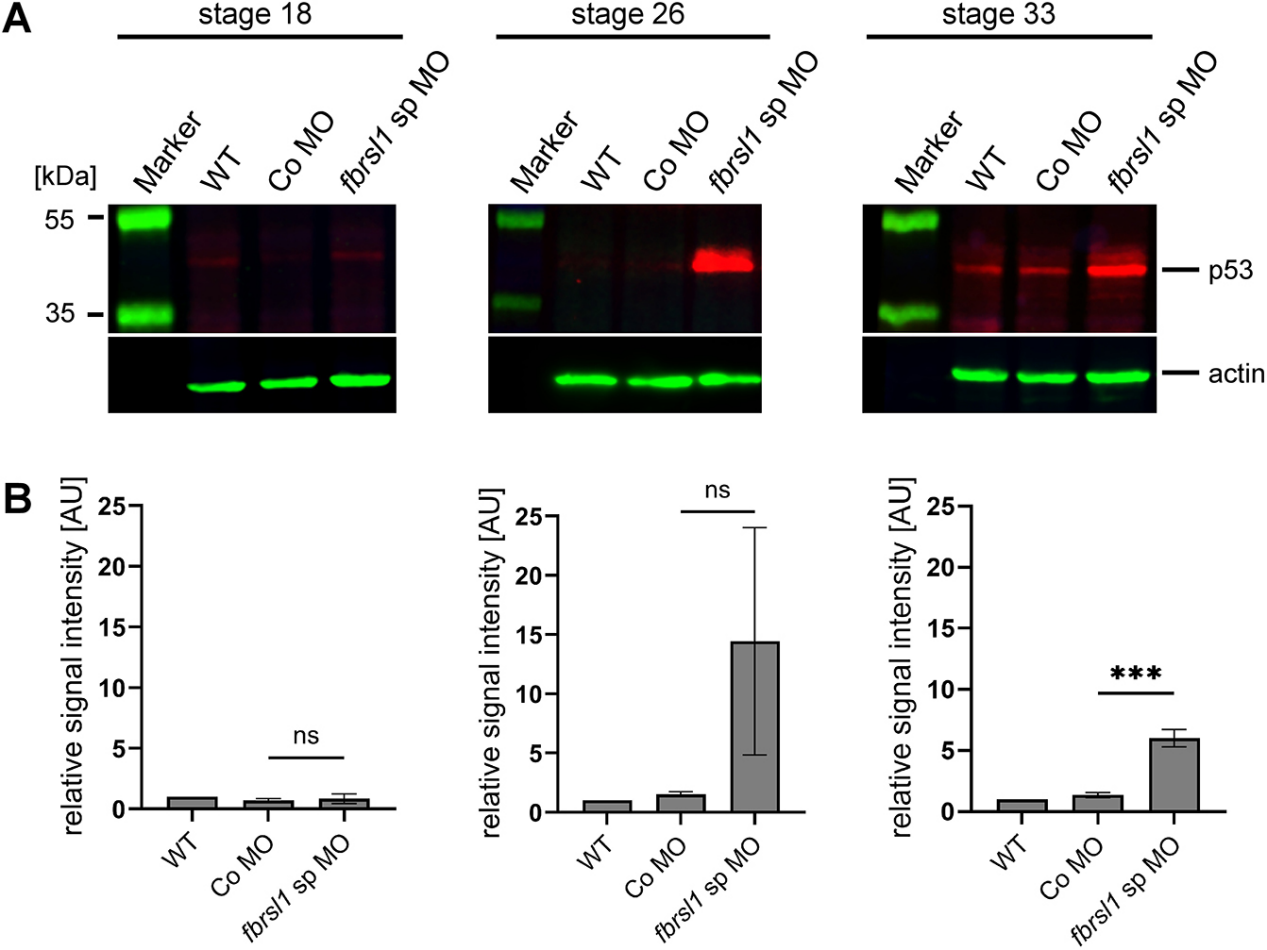
**p53 protein levels are increased in *fbrsl1* morphant tadpole embryos.** One-cell stage *Xenopus* embryos were injected animally with 20 ng of Co MO or *fbrsl1* sp MO together with 100 pg *GFP* RNA or 0.25 µg/µl fluorescein-dextran for lineage tracing. (A) p53 protein levels were analyzed by western blotting at stage 18, stage 26 and stage 33. Representative western blots are shown. Molecular masses (kDa) are indicated. (B) The p53-to-actin signal intensity ratio for all samples was normalized to that of uninjected controls (WT). The graphs summarize three independent experiments. Data are mean±s.e.m.; ns, not significant; ***P≤0.001 (one-way ANOVA with Tukey's multiple comparison test).

At late stages of NC migration, *fbrsl1* morphants lose the expression of NC markers and transcription factors required for craniofacial development. Furthermore, explanted *fbrsl1* morphant NC cells, although they showed normal migration and dispersion (Fig. 3), started to round up and die earlier than controls (Movie 1). Therefore, we analyzed whether these defects may be caused by apoptosis. The expression of p53 was analyzed at different stages of NC migration in embryos injected either with Co MO or *fbrsl1* sp MO using western blotting (Fig. 5). At stage 18 (early migration), p53 expression was very low in *fbrsl1* morphants and controls. At stage 26, we noted an increase in p53 expression compared to controls, which became statistically significant by stage 33 (late migration) (Fig. 5A,B).

**Fig. 6.**
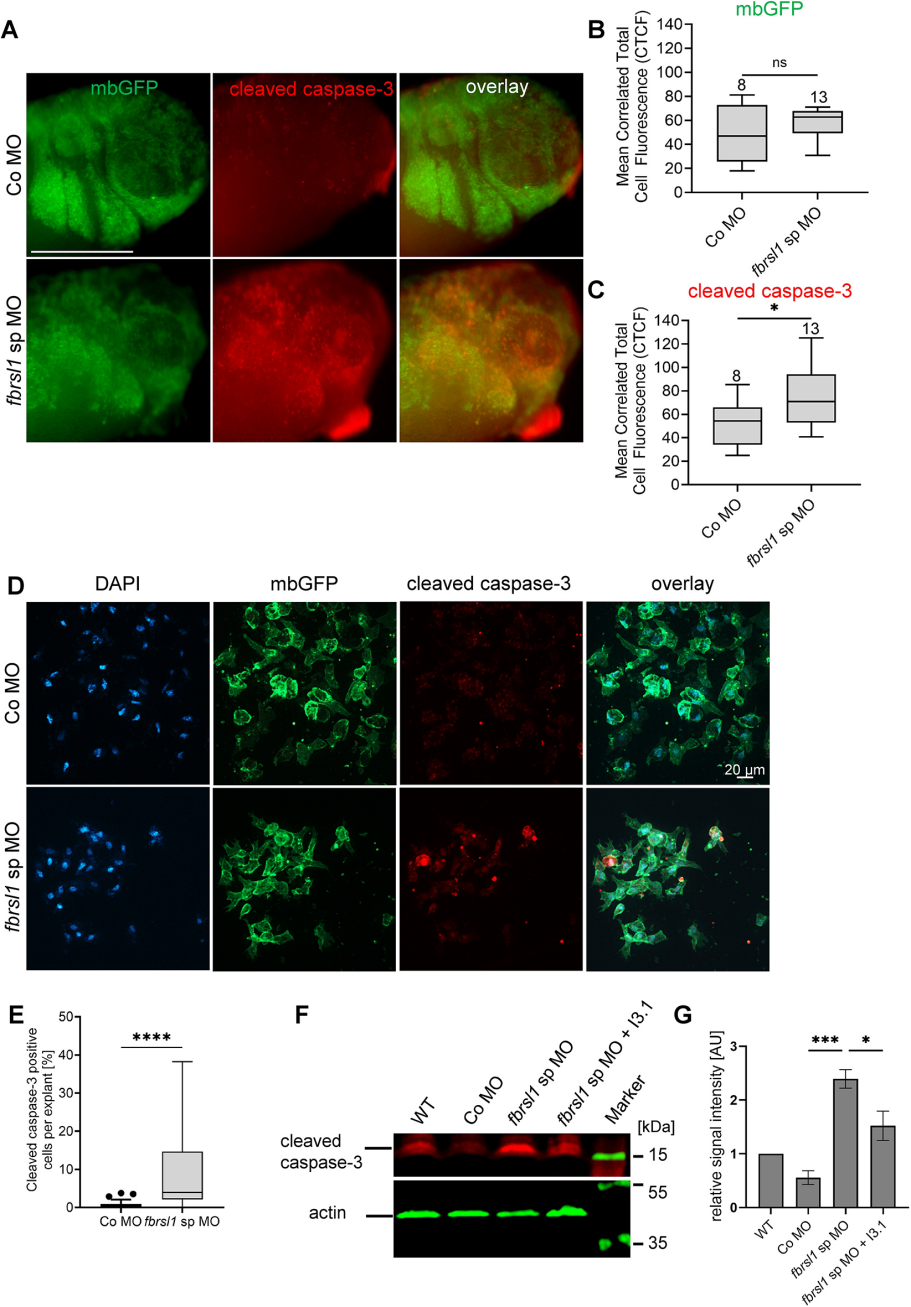
**Cleaved caspase-3 signal intensity is increased in the branchial arch area of Fbrsl1-depleted embryos and in explanted NC cells.** (A-C) *Xenopus* embryos were injected with 10 ng of Co MO or *fbrsl1* sp MO, together with 100 pg *mbGFP* RNA for lineage tracing in one blastomere at the two-cell stage. Embryos were analyzed by immunostaining for cleaved caspase-3 at stage 31/32. (A) Co MO-injected embryos showed weak cleaved caspase-3 expression, which was increased in embryos injected with the *fbrsl1* sp MO. Cleaved caspase-3 expression localized to the area of cranial NC migration. Scale bar: 500 µm. (B,C) Boxplots with Tukey's whiskers set at 1.5 times the IQR summarize data from three independent experiments analyzing CTCF intensity of mbGFP (B) or cleaved caspase-3 (C). The median is indicated by a line within the box; the boxes extend from the 25th to the 75th percentile. The number of embryos analyzed is indicated above each box; ns, not significant; *P≤0.05 (unpaired Student's *t*-test). (B) CTCF intensity of mbGFP does not significantly change between Co MO and *fbrsl1* sp MO-injected embryos. (C) Cleaved caspase-3 CTCF intensity is significantly increased in embryos injected with *fbrsl1* sp MO in comparison to Co MO-injected embryos. (D) NC explants of embryos were injected with 10 ng of Co MO or *fbrsl1* sp MO, together with 150 pg *mbGFP* RNA. GFP and cleaved caspase-3 expression was analyzed by immunostaining. (E) Graph showing the percentage of cleaved caspase-3-expressing cells in *fbrsl1* morphant and control NC cells of three independent experiments. ****P≤0.0001 (Mann–Whitney test). Boxplots with Tukey's whiskers set at 1.5 times the IQR; dots represent outliers; the median is indicated by a line within the box; the boxes extend from the 25th to the 75th percentile). (F) *Xenopus* embryos were injected with 20 ng of Co MO or *fbrsl1* sp MO, together with 100 pg *GFP* RNA in the animal pole at the one-cell stage. For rescue experiments, 400 pg human HA-FBRSL1-I3.1-pcDNA3.1 (I3.1) was co-injected. The levels of cleaved caspase-3 were analyzed by western blotting at stage 33. A representative western blot is shown. Molecular masses (kDa) are indicated. (G) The ratio of signal intensity levels of cleaved caspase-3 to actin was normalized to wild-type cleaved caspase-3 levels and plotted. The graph summarizes three independent experiments. Data are mean±s.e.m. Cleaved caspase-3 protein levels significantly increased in Fbrsl1-depleted embryos. This effect was partially rescued by co-injection of I3.1. *P≤0.05, ***P≤0.001 (one-way ANOVA with Tukey's multiple comparison test).

To confirm this finding, we also analyzed the expression of cleaved (active) caspase-3, another apoptosis marker. Embryos were injected with MOs and *mbGFP* RNA as a lineage tracer in one blastomere at the two-cell stage and cleaved caspase-3 expression was analyzed by immunostaining at stage 31/32. Prior to imaging, the epidermis was removed to prevent cleaved caspase-3 staining in epidermal cells from obscuring the signal in NC cells. Interestingly, this procedure revealed a strong GFP signal in the branchial arch region, which facilitated the identification of cleaved caspase-3-positive cells within this area. In control embryos, we detected a weak expression of cleaved caspase-3, which significantly increased in *fbrsl1* morphant embryos (Fig. 6A). Interestingly, the fluorescence was most intense in the branchial arches, which are populated by migrating cranial NC cells. This increase in fluorescent intensity was also confirmed by measuring the mean correlated cell fluorescence (CTCF), which was significantly increased, while the CTCF for mbGFP fluorescence was unaffected by loss of Fbrsl1 function (Fig. 6B,C). Furthermore, to assess whether there is a significant increase in cleaved caspase-3 in NC cells, we additionally analyzed explanted NC cells. Embryos were injected at the two-cell stage with *fbrsl1* sp MO and *mbGFP* RNA. Premigratory NC cells were explanted, cultured to late migratory stages, when they were fixed and analyzed by immunostaining for cleaved caspase-3. Consistent with the analysis in whole embryos, a significant increase in cleaved caspase-3-positive cells was observed in *fbrsl1* morphant NC cells (Fig. 6D,E). These data suggest that loss of Fbrsl1 leads to apoptosis of NC cells.

To confirm that the increase in apoptosis markers is specific to loss of Fbrsl1 function, we performed rescue experiments. Embryos were injected with MOs in combination with fluorescein-dextran as a lineage tracer. For rescue experiments, the short N-terminal FBRSL1 isoform I3.1 was co-injected. The expression of cleaved caspase-3 was analyzed by western blotting at stage 33. Consistent with the immunostaining experiments in embryos, we detected a significant increase in the expression of cleaved caspase-3 in embryos injected with the *fbrsl1* sp MO compared to controls (Fig. 6F,G). Similar effects were observed for *fbrsl1* tb MO (Fig. S4). Co-expression of I3.1, which is able to partially rescue the craniofacial defects of *fbrsl1* morphant embryos (Ufartes et al., 2020), was also able to partially rescue the increase in cleaved caspase-3 expression observed in *fbrsl1* morphants (Fig. 6F,G). Taken together, these data indicate that loss of Fbrsl1 function leads to p53-mediated apoptosis of NC cells at late stages of migration.

**Fig. 7.**
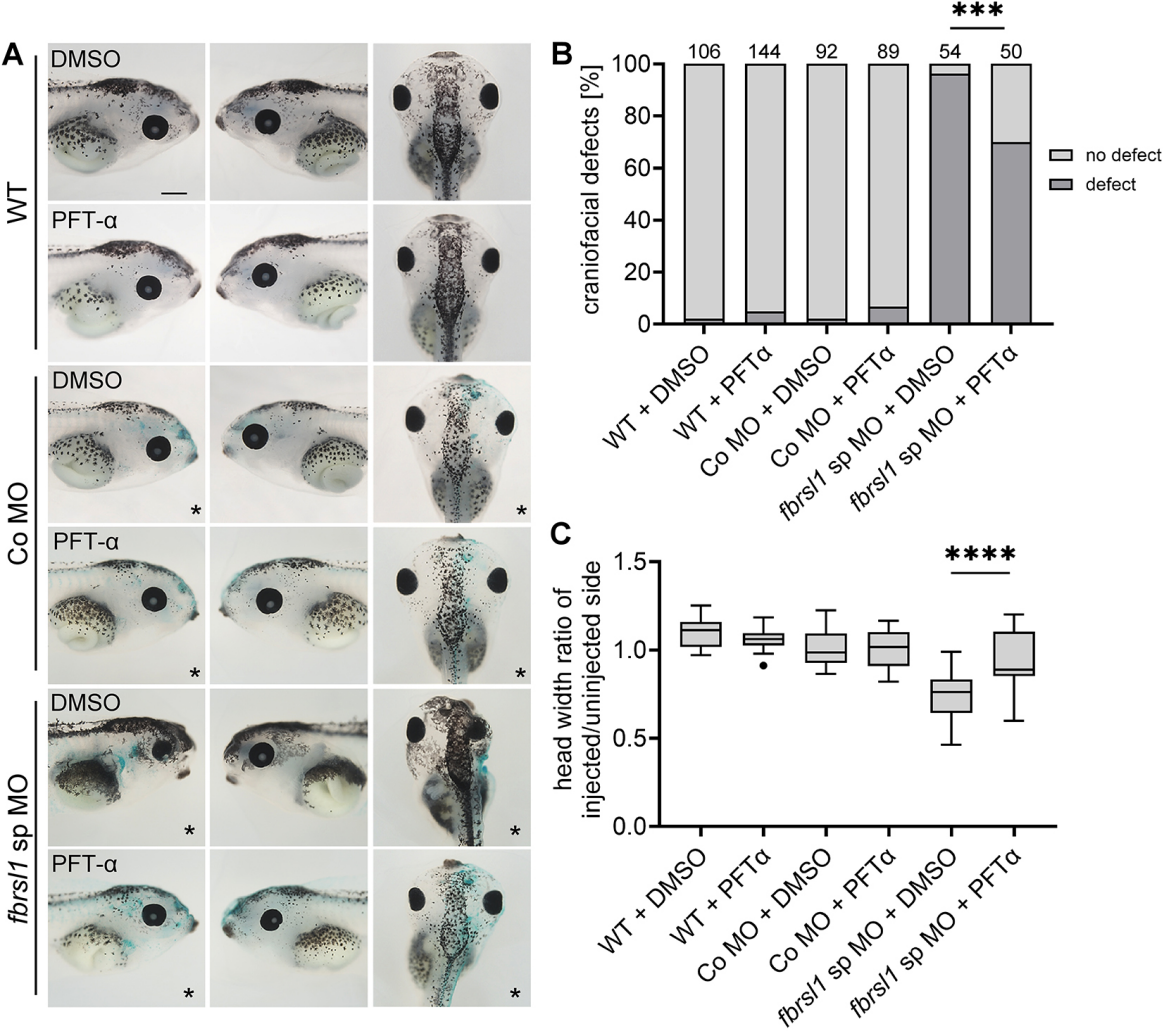
**PFT-α treatment partially rescues craniofacial defects induced by Fbrsl1 depletion.** Embryos were injected with 10 ng Co MO or 10 ng *fbrsl1* sp MO, in combination with 100 pg *lacZ* RNA in one-blastomere at the two-cell stage. From stage 20 to stage 33, *Xenopus* embryos were incubated in 20 µM of the p53 inhibitor PFT-α (pifithrin-α) or in 0.1% DMSO as solvent control. PFT-α incubation results in a partial rescue of craniofacial defects induced by the injection of the *fbrsl1* sp MO. PFT-α or DMSO incubation did not affect development of 10 ng Co MO-injected embryos. Dorsal views of representative embryos are shown. The injected side is marked with an asterisk. Scale bar: 500 µm. (B) Data in the graph are presented as the percentage of the total, based on three independent experiments; the number of analyzed embryos are indicated; ***P≤0.001 (two-sided Fisher's exact test). (C) Quantification of the ratio of the head width of the injected side in comparison to the uninjected side of 20 randomly selected embryos. ****P≤0.0001 (one-way ANOVA with Tukey's multiple comparison test). Boxplots with Tukey's whiskers set at 1.5 times the IQR, dots represent outliers; the median is indicated by a line within the box; the boxes extend from the 25th to the 75th percentile.

If p53-mediated apoptosis of NC cells contributes to the craniofacial phenotype observed in *fbrsl1* morphants, then inhibition of p53 signaling may rescue the morphant phenotype. To test whether this is the case, we injected embryos with MOs and incubated them either with the p53 inhibitor pifithrin-α (PFT-α) (Komarov et al., 1999) or a solvent control from stage 20 to stage 33. As a control, uninjected embryos were treated in the same way. Craniofacial defects were scored as visible abnormalities in head size, head shape and eye asymmetries compared to stage-matched controls (Fig. 7). Consistent with our hypothesis, we observed a significant although partial rescue effect in *fbrsl1* morphant embryos that were treated with PFT-α compared to the solvent control (Fig. 7A,B). A similar effect was observed, when craniofacial defects were quantified by determining the ratio of the head width of the injected side in comparison to the uninjected side (Fig. 7C). Taken together, these data indicate that p53-mediated apoptosis contributes to the observed defects in NC development.

### Fbrsl1 loss of function causes exon-skipping in *mdm2*

**Fig. 8.**
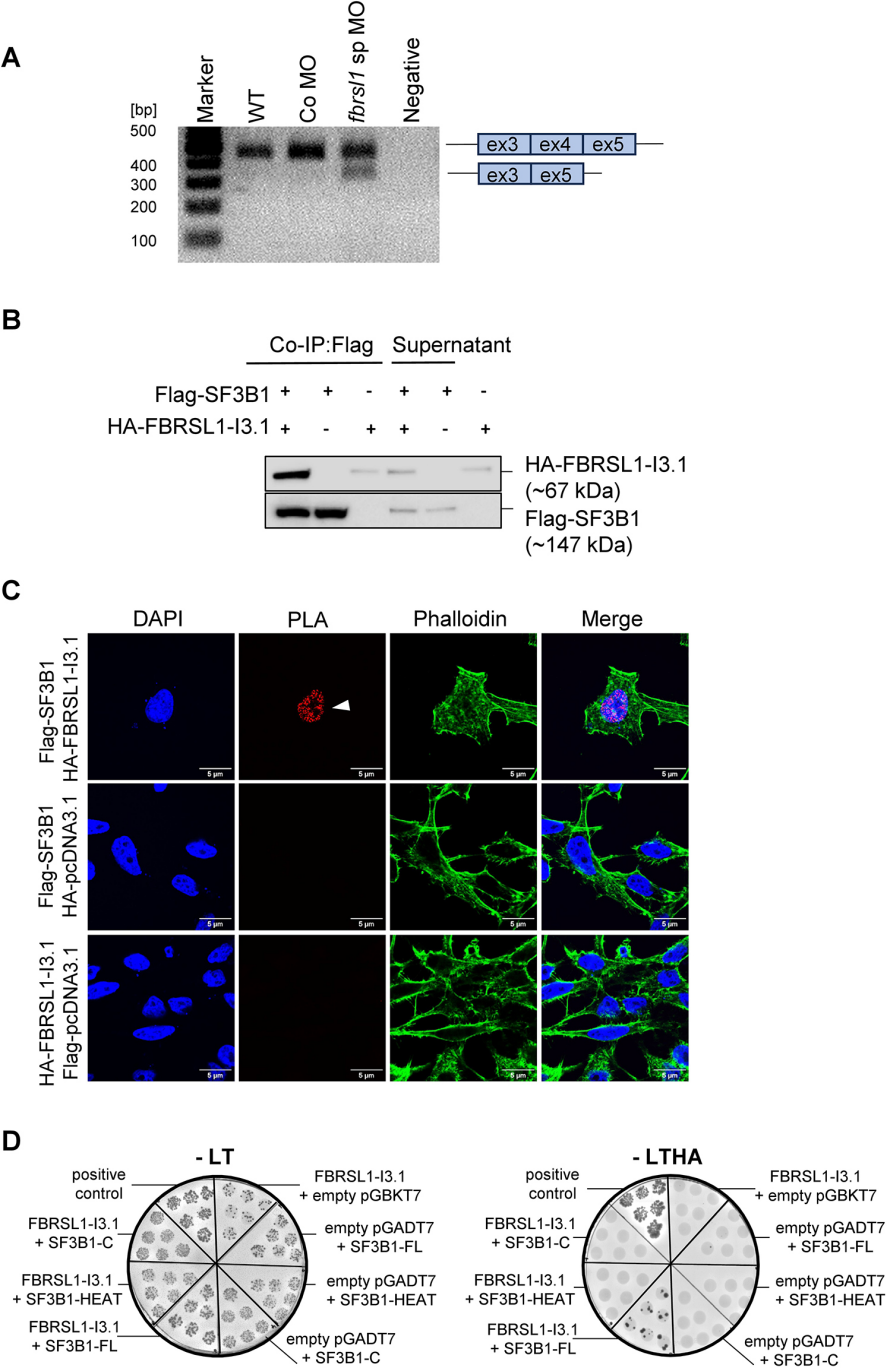
**FBRSL1-I3.1 interacts with the splicing factor SF3B1 and loss of Fbrsl1 function leads to increased exon 4 skipping in *mdm2*.** (A) Embryos were injected with 20 ng Co MO or *fbrsl1* sp MO at the one-cell stage, and RNA was isolated at stage 33 and reverse-transcribed into cDNA. PCR followed by agarose gel electrophoresis revealed an increased exon 4 skipping of *mdm2* in *Xenopus* embryos with *Fbrsl1* knockdown in comparison to wild-type (WT) or Co MO-injected embryos. ex, exon. (B) HEK293 cells were co-transfected with Flag-SF3B1 and HA-FBRSL1-I3.1. Co-immunoprecipitation was performed using an anti-Flag antibody, and precipitated proteins were detected by western blotting. Co-IP, co-immunoprecipitation. (C) Proximity ligation assay (PLA) showing the localization of the FBRSL1-SF3B1 association. Positive PLA signals (indicated by the white arrowhead) were observed after co-transfection of Flag-SF3B1 with HA-FBRSL1-I3.1, indicating an interaction in the nucleus. Co-transfection with empty vectors (Flag-pcDNA3.1 and HA-pcDNA3.1) served as negative controls. DNA was counterstained with DAPI, and the actin cytoskeleton was visualized using phalloidin. The images for Flag-SF3B1 HA-pcDNA3.1 are also shown in Fig. S6. (D) A yeast two-hybrid (Y2H) experiment revealing a direct interaction of FBRSL1 isoform I3.1 with SF3B1. A representative Y2H experiment showing colonies on the −LTHA selective plate after co-transformation of FBRSL1-I3.1 with full-length (FL) SF3B1, indicating a direct interaction. No colonies were observed after co-transformation with a C-terminal SF3B1 construct (SF3B1-C, amino acids 1100-1304) or the HEAT domain of SF3B1 (SF3B1-HEAT, amino acids 501-1304). The −leucine/−tryptophan (−LT) selective plates served as a control for co-transformation efficiency. Co-transformation of KMD6A with KMT2D-pt.8 served as a positive control. Auto-activation of bait and prey proteins was tested by co-transforming the constructs with the corresponding empty vector.

Upregulation of p53 is a well-known mechanism of human congenital disorders that affect craniofacial development and, reminiscent of our findings in *fbrsl1 Xenopus* morphants, loss of p53 function can at least to some extent rescue the disease phenotype in mouse model systems (Bowen and Attardi, 2019; Tsai et al., 2021; Van Nostrand et al., 2014). For craniofacial spliceosomopathies, which are developmental disorders caused by haploinsufficiency of a component of the spliceosome machinery (Beauchamp et al., 2020; Griffin and Saint-Jeannet, 2020), it is suggested that inefficient splicing results in increased skipping of regulatory exons in *MDM2* in NC cells (Beauchamp et al., 2020). As a consequence, p53 is upregulated, leading to NC cell apoptosis. Reminiscent of the malformations observed in patients affected by the FBRSL1-associated syndrome, patients with craniofacial spliceosomopathies show microcephaly, intellectual disability, heart defects and craniofacial malformations (Beauchamp et al., 2020). Therefore, we analyzed whether *mdm2* splicing is also affected in *fbrsl1* morphant embryos. To this end, embryos were injected with the *fbrsl1* sp MO or control MO at the one-cell stage, in combination with *mbGFP* RNA, and *mdm2* expression was analyzed at stage 33 by RT-PCR. Indeed, a smaller *mdm2* band was detected by using primers spanning exon 1 to exon 5 (Fig. 8A), with a markedly stronger signal in *fbrsl1* morphants compared to controls. Sanger sequencing confirmed that this transcript was generated by skipping of exon 4 of *mdm2* (Fig. S5). Thus, loss of Fbrsl1 function seems to result in an increased exon 4 skipping of *mdm2*, likely leading to subsequent activation of p53.

### FBRSL1 interacts with the splicing factor SF3B1

The mechanism by which Fbrsl1 loss of function leads to mis-splicing of *mdm2* is currently unknown. Since it was previously shown that the FBRSL1 paralog AUTS2 interacts with the splicing factor and core member of the splicesome SF3B1 (Monderer-Rothkoff et al., 2021), we investigated whether FBRSL1 itself may also influence splicing through interaction with the splicing machinery. To address this, we performed co-immunoprecipitation experiments to analyze whether FBRSL1 also interacts with SF3B1. HEK293 cells were co-transfected with Flag-tagged SF3B1 (Flag-SF3B1) in combination with the HA-tagged long isoform (HA-FBRSL1-I1) or the short FBRSL1 isoform (HA-FBRSL1-I3.1). SF3B1 was immunoprecipitated using an anti-Flag antibody and the co-precipitated FBRSL1 proteins were detected by western blotting using an anti-HA antibody. Protein bands of ∼112 kDa, corresponding to the expected size of HA-FBRSL1-I1 (Fig. S6A), and of ∼67 kDa, corresponding to the expected size of HA-FBRSL1-I3.1 (Fig. 8B), were detected, indicating that SF3B1 interacts with the different FBRLS1 isoforms.

To further validate and map the interaction between FBRSL1 and SF3B1, we performed an *in situ* proximity ligation assay (PLA) in HeLa cells co-transfected with Flag-SF3B1 in combination with HA-FBRSL1-I3.1 or HA-FBRSL1-I1 (see Fig. 8C or Fig. S6B, respectively). Co-transfection of empty vectors served as controls. Indeed, immunofluorescence analysis revealed distinct nuclear PLA signals after co-transfection of SF3B1 with both FBRSL1 isoforms, confirming the interaction shown by Co-IP.

Since the FBRSL1 paralog AUTS2 was shown to directly interact with SF3B1 (Monderer-Rothkoff et al., 2021), we analyzed whether the long or the short N-terminal isoform of FBRSL1 might also directly interact with SF3B1. To this end, we performed yeast two-hybrid (Y2H) assays with both FBRSL1 isoforms and full-length SF3B1 (SF3B1-FL) (Fig. 8D). To further delineate potential binding regions within SF3B1, additional Y2H assays were conducted testing only the HEAT domain (amino acids 501-1304; SF3B1-HEAT) and the C-terminal region (amino acids 1100-1304, SF3B1-C) of SF3B1 (Fig. S6C). Interestingly, yeast growth on -LTHA selective plates was observed only after co-transformation of full-length SF3B1 with the short N-terminal FBRSL1 isoform I3.1 (Fig. 8D, Fig. S5D). These observations indicate a direct interaction of the FBRSL1 isoform I3.1 with SF3B1, while the isoform I1 is likely associated with SF3B1 via an indirect mechanism. Since no yeast growth was observed after co-transformation with either the HEAT domain or the C-terminal region of SF3B1 (Fig. 8D, Fig. S5C), the binding site of FBRSL1-I3.1 is suspected to reside within the N-terminal region of SF3B1, or the interaction may require proper protein folding that may occur only in the full-length SF3B1 protein. Collectively, our protein-protein interaction analyses indicate that both long and N-terminal FBRSL1 isoforms associate with SF3B1 in the nucleus, with the N-terminal isoform I3.1 showing a direct interaction. Taken together, these data suggest a model in which loss of Fbrsl1 may impact Sf3b1-dependent splicing, thereby causing p53-mediated apoptosis of cranial NC cells destined for cartilage formation.

## DISCUSSION

All currently identified patients with truncating *FBRSL1* variants exhibit, among other features, craniofacial malformations of varying severity. The data presented here support the classification of the FBRSL1-associated syndrome as a neurocristopathy – a group of congenital disorders arising from defects at various stages of NC development (Bolande, 1974). While NC induction, specification and early stages of *Xenopus* NC positioning are largely unaffected by Fbrsl1 loss of function, significant defects were observed at late stages of NC migration. Interestingly, these seem to be independent of the ability of NC cells to migrate, as protrusion formation, velocity or persistence of migration are normal. Nevertheless, defects were seen in NC cells fated to contribute to craniofacial structures. The loss of marker expression – or possibly even the loss of cells expressing these markers – correlates with an increase in p53/cleaved caspase-3 expression, while cell proliferation seemed not to be affected (Fig. S7). This observed increase in apoptosis markers is unlikely to be the result of morpholino toxicity, because it is not seen until late stages of NC migration, it is independent of the used *fbrsl1* MO (splice blocking versus translation blocking) and it can be partially rescued by the human N-terminal FBRSL1 isoform I3.1. We currently do not know whether the increase in p53 expression is NC specific, but the increased expression of cleaved caspase-3 in explanted *fbrsl1* morphant NC cells suggests that the NC is at least among the tissues that are targeted by p53-mediated apoptosis.

Upregulation of p53 is a well-known mechanism in neurocristopathies, such as CHARGE and Treacher Collins syndrome, as well as in spliceosomopathies like Richieri-Costa-Pereira (OMIM268305), cerebrocostomandibular (OMIM117650) and thrombocytopenia-absent radius (TAR) syndrome (OMIM274000) that also show craniofacial defects (Beauchamp et al., 2020; Tsai et al., 2021; Van Nostrand et al., 2014). These syndromes are often referred to as p53 hyperactivity syndromes because – as we have seen this for *fbrsl1* morphants – malformations can be rescued by reducing the p53 levels in the respective model systems (Van Nostrand et al., 2014). Although the molecular mechanisms by which p53 is activated are diverse, spliceosomopathies that affect craniofacial development show interesting parallels to our results. For example, variants in the splicing factor *EFTUD2* that cause mandibulofacial dysostosis with microcephaly seem also to affect the splicing of *MDM2*. This is supported by findings in mice, where homozygous deletion of *Eftud2* increased exon skipping and resulted in alternatively spliced *Mdm2* transcript lacking exon 3 (Beauchamp et al., 2021). Mis-splicing of *Mdm2* produces a truncated protein that can no longer bind to MDM4, thereby leading to stabilization and increased levels of p53 (Giglio et al., 2010). Consistent with defects in *Mdm2* splicing, nuclear p53 levels and cell death were increased. Similar to our findings in *fbrsl1* morphants, treatment with the p53 inhibitor pifithrin-α rescued craniofacial defects in *Eftud2* mutants (Beauchamp et al., 2021). In line with this, knockdown of *SF3B1*, a core member of the SF3B spliceosome complex, led to an increase in p53 levels caused by exon skipping in *MDM2* (Allende-Vega et al., 2013). Heterozygous variants of *SNRPB*, a core component of the five small ribonucleoprotein particles of the spliceosome, cause cerebrocostomandibular syndrome. Heterozygous deletion of *Snrpb* in mice increased exon skipping of negative regulators of the p53 pathway, p53 upregulation and apoptosis (Alam et al., 2022). Again, exon 3 skipping of *Mdm2* was observed. Interestingly, this study also identified a large number of genes that were mis-spliced, including several genes required for craniofacial development (Alam et al., 2022). Thus, variants in the splicing machinery may impact signaling networks relevant for craniofacial development at multiple levels.

The *fbrsl1* morphant phenotype may also be caused by global defects in splicing. Support for this hypothesis comes from the finding that FBRSL1 interacts with SF3B1, a key component of the SF3B complex. Thus, loss of function could affect the activity of SF3B1, thereby affecting splicing of multiple targets. The importance of the SF3B complex components in NC development has been demonstrated in different model systems. For example, hypomorphic *Sf3b1* mutant zebrafish embryos show defects in NC lineage specification, survival and migration, resulting in reduced melanocyte numbers and craniofacial abnormalities. Sequence analysis of selective NC regulators showed that the pre-mRNA processing of *sox9b* and *snai1b* transcripts was severely disrupted in mutants compared to controls (An and Henion, 2012). Furthermore, homozygous deletion of *Sf3b4* in mouse NC cells, which caused craniofacial and cardiac malformations, resulted in mis-splicing and reduced expression of NC specifiers and histone modifiers important for craniofacial and heart development (Kumar et al., 2024). Recently, homozygous deletion of *sf3b4* in *Xenopus tropicalis* was shown to severely impair the development of NC-derived cartilage progenitors (Griffin et al., 2025). Interestingly, these mutants resemble *fbrsl1* morphant embryos in that they show defects at late stages of NC migration, while NC induction was unaffected. As observed in *fbrsl1* morphants, the expression of markers associated with NC-derived cartilage formation – and cartilage development itself – was significantly impaired and an increase in apoptosis in the head region was observed. Since NC migration was not analyzed in detail, it is unclear if this process is affected in *sf3b4* mutants. Another interesting parallel to *fbrsl1* morphants is that *sf3b4* null mutants also show cardiac defects (Berger et al., 2024; Griffin et al., 2025). Consistent with the observation that developmental defects became more severe from tailbud to tadpole stages, RNA sequencing analysis revealed a greater number of dysregulated genes at the tadpole stage, including genes relevant for splicing, apoptosis and neural crest migration, compared to tailbud or neurula stages (Griffin et al., 2025). Interestingly, exon-skipping events were more frequently observed at earlier stages, likely preceding or possibly even causing the changes in gene expression. These data suggest that some aspects of the *fbrsl1* morphant phenotype could potentially be linked to defects in splicing, although at this stage this remains a hypothesis that requires further experimental validation. Taken together, our data add the FBRSL1-associated syndrome to the list of neurocristopathies, but it remains to be seen whether it is also a spliceosomopathy.

## MATERIALS AND METHODS

### *Xenopus laevis* microinjection and inhibitor treatment

*Xenopus laevis* stimulation of ovulation as well as testis preparation to obtain fertilized eggs were performed according to the German animal use and care law (Tierschutzgesetz) and approved by the German state administration Hesse (Regierungspräsidium Gießen MR 17/1 Nr. V7/2022). *Xenopus* embryos were obtained and cultured according to standard protocols, and staged based on the normal table of Nieuwkoop and Faber (Nieuwkoop and Faber, 1956). *Xenopus* embryos were injected in one blastomere at the two-cell stage with morpholino oligonucleotides (MO) (Gene Tools), except for western blotting and RT-PCR experiments, where embryos were injected at the one-cell stage. For Fbrsl1 loss of function, either a splice-blocking *fbrsl1* MO (*fbrsl1* sp MO; Ufartes et al., 2020), which blocks the splicing of the first intron, or a translation-blocking MO (*fbrsl1* tb MO; Berger et al., 2024) were used. A standard control MO (Co MO: 5′-CCT CTT ACC TCA GTT ACA ATT TAT A-3′) (Gene Tools) served as a control; *lacZ* RNA (Smith and Harland, 1991), *mbGFP* RNA (Moriyoshi et al., 1996) or 0.25 µg/µl fluorescein-dextran (Invitrogen) were co-injected for lineage tracing. Capped sense mRNAs were synthetized using the mMessage mMachine SP6 Transcription Kit (Invitrogen). For rescue experiments, plasmids encoding human FBRSL1 isoform 1, isoform 3.1 and the variant isoform 3.1-p.Q163* (Ufartes et al., 2020) were co-injected together with the *fbrsl1* sp MO. The concentrations of injected morpholino oligonucleotides, RNA and plasmids are indicated in the figure legends.

For p53 inhibitor treatment, *Xenopus* embryos were incubated in 20 µM pifithrin-α (PFT-α; Santa Cruz, sc-45050) (Komarov et al., 1999) dissolved in DMSO or a 0.1% DMSO solvent control diluted in 0.1× MBS from stage 20 until stage 33. Following PFT-α treatment, the embryos were washed in 0.1× MBS and incubated until stage 43 for craniofacial development analysis.

### Whole-mount *in situ* hybridization of *Xenopus* embryos

To analyze NC marker expression, embryos were fixed in MEMFA (3.7% formaldehyde, 0.1 M MOPS, 2 mM EGTA and 2 mM MgSO_4_) at the indicated stages and β-galactosidase staining and *in situ* hybridization were performed according to standard protocols (Harland, 1991; Smith and Harland, 1991). The following constructs were used to prepare *in situ* hybridization probes: *pax3* (Espeseth et al., 1995), *snai1* (Mayor et al., 1995), *twist* (Hopwood et al., 1989), *sox9* (Spokony et al., 2002) and *gsc* (Cho et al., 1991).

To generate a *dlx5* antisense *in situ* hybridization probe, a 870 bp *dlx5* fragment was amplified by PCR from *Xenopus laevis* cDNA using the following primers: 5-TATAATCGATATGACAGGAGTCTATGAGCGGA-3 and 5-TATACTCGAGTTAGTAGAGAGTCCCTGATGCC-3. The PCR product was cloned into the pCS2+ vector using ClaI and XhoI restriction enzyme sites.

### Imaging and statistical analysis

Embryos were imaged using a Nikon stereo microscope (SMZ18) with a DS-Fi3 Nikon camera and NIS-Elements imaging software. The investigators were blinded to the group allocation when they assessed the experimental outcome. Phenotypes analyzed by *in situ* hybridization were categorized according to the classification scheme presented in Fig. S2. To quantify the expression defects, the area of the *twist* or *sox9* expression was measured manually by using the Fiji (ImageJ) version 1.54f (Schindelin et al., 2012) polygon selection tool and the ratio between the injected and uninjected side was determined. For the quantification of craniofacial defects, the ratio of the head width of the injected side in comparison to the uninjected side was determined using the straight-line tool in Fiji. Statistical analysis was performed using GraphPad PRISM Software (Graphpad Software) with the indicated *P* values: *P≤0.05, **P≤0.01, ***P≤0.001 and ****P≤0.0001. Statistical tests were selected based on the characteristics of the raw data and are indicated in the figure legends.

### NC explants, transplants and live-cell imaging

NC cells were transplanted as previously described (Borchers et al., 2000). In brief, the fluorescent pre-migratory NC cells were cut out at stage 18 and transplanted into wild-type host embryos from which the NC cells had been removed. NC migration was documented at stage 28 using the Nikon stereo microscope (SMZ18) with a DS-Fi3 Nikon camera and NIS-Elements imaging software. The length of the migration streams, defined as the distance from the back of the embryo to the tip of the migration front, was measured using the straight-line tool of Fiji.

To analyze NC migration at the cellular level, embryos were injected with MOs in combination with 150 pg *mbGFP* mRNA to mark the cell membrane and 250 pg *H2B mCherry* mRNA to visualize the nucleus. The NC explants were dissected at stage 18 and cultured in 0.8× MBS on fibronectin-coated (10 µg/ml, F1141-5MG, Sigma Aldrich) chamber slides. Live-cell imaging of NC cell explants was performed using spinning disk confocal microscopy (Axio Observer Z1, Zeiss).

The analysis of protrusion formation was conducted approximately 2-3 h after dissection using a 63× oil objective. The circularity of NC cells was analyzed manually, using the polygon selection tool and shape descriptor measurements in Fiji. To analyze the migration behavior over time, cells were recorded for 5 h with a 10× objective. The dispersion of NC cells was analyzed by Delaunay triangulation using the dispersion tool in Fiji (plugin kindly provided by Roberto Mayor). Explants were quantified using the mean triangle size between individual nuclei of NC cells for each explant after 5 h of cultivation (Barriga et al., 2019). These recordings were additionally analyzed in Imaris to measure the cell velocity and persistence of migration. Therefore, only cells that migrated without contact with other cells for at least 1 h were selected for the analysis.

For immunostaining of explanted NC cells, embryos were injected with the respective MOs in combination with 100-150 pg *mbGFP* mRNA. Cells were treated as described above and fixed in MEMFA for 20 to 30 min, ∼7-8 h after dissection for cleaved caspase-3 immunostaining or 4-6 h after dissection for PH3 immunostaining. Immunostaining was performed as previously described (Gossen et al., 2024), except that blocking was carried out for 30 min to 1 h at room temperature. The primary antibodies used were anti-active caspase-3 (570524 BD Pharmingen, 1:250), anti-GFP (11814460001 Roche, 1:500) and anti-PH3 (phosphor-histone H3; 05-806 Sigma Aldrich, 1:500). The secondary antibodies used were anti-rabbit Alexa 488 (A21206 Life Technologies, 1:400), anti-rabbit Alexa 594 (A11012 Life Technologies, 1:400), anti-mouse Alexa 488 (A11029 Life Technologies 1:400) and anti-mouse Alexa 594 (A11005 Life Technologies 1:400). The cells were fixed on microscope slides with mounting medium (Dako) supplemented with DAPI (Sigma, 1 µg/ml). Imaging was performed using spinning disk confocal microscopy (for cleaved caspase-3) or a Leica TCS SP5 confocal microscope (for PH3). The caspase-positive cells and the total number of cells were counted using the multi-point tool of Fiji. The PH3-positive cells and the total number of cells were counted with the automated particle counter of Fiji.

### RNA isolation, cDNA synthesis and RT-PCR

Total RNA was isolated from 10-20 stage 33 embryos that had been injected at the one-cell stage with 20 ng of either Co MO or *fbrsl1* sp MO, as well as from uninjected controls, using the Illustra RNAspin Mini Isolation Kit (GE Healthcare). 1 ng RNA per sample was reverse transcribed into complementary DNA (cDNA) using the Tetro cDNA synthesis kit (Meridian Bioscience). PCR amplification of *mdm2* transcripts was performed using the following primers: 5′-AGCCGAAAACGCAGGAATG-3′ and 5′-CTAGAAATCATTGCATACAGTCTC-3′. Agarose gel electrophoresis was performed to separate PCR products. The PCR products of *Mdm2* were purified from the agarose gel using the NucleoSpin Gel and PCR Clean-up kit (Macherey-Nagel), and sequenced by Microsynth Seqlab.

### P53, cleaved caspase-3 and PCNA expression

For western blotting, *Xenopus* lysates were prepared as previously published (Berger et al., 2024). For immunodetection, the following primary antibodies were used: p53 (ab16465, Abcam and MA1-12549, Invitrogen, 1:250), caspase-3 (9662, Cell Signaling, 1:500), PCNA (PC10, Santa Cruz, sc-56, 1:1000) and anti-Actin (MAB1501, Merck Millipore, 1:2000). As secondary antibodies we used IRDye 800CW donkey anti-rabbit IgG secondary antibody (P/N: 926-32213, LI-COR, 1:7500) and IRDye 680RD donkey anti-mouse IgG secondary antibody (P/N 926-68072, LI-COR, 1:7500). Proteins were detected using the Odyssey Fc Imaging System (LI-COR Bioscience).

For whole-mount immunostaining, *Xenopus* embryos were incubated until stage 31/32, fixed with Dent's fixative (20% DMSO, 80% methanol) and whole-mount immunofluorescence staining was performed as previously described (Ufartes et al., 2020). The primary antibodies used were anti-active caspase-3 (570524 BD, Pharmingen, 1:500) and anti-GFP (ab290 Abcam, 1:500); the secondary antibodies were anti-rabbit Alexa 488 (A21206 Life Technologies, 1:400) and anti-mouse Alexa 594 (A11005 Life Technologies 1:400). For imaging, the epidermis was removed to prevent cleaved caspase-3 staining in epidermal cells from obscuring the signal in NC cells. Fluorescence intensity was quantified by calculating the corrected total cell fluorescence (CTCF) (Gavet and Pines, 2010) using Fiji. To determine sample fluorescence, a standardized rectangle of equal size was placed over the region exhibiting active NC cell migration in each embryo. Background fluorescence was calculated as the average signal from three randomly selected areas outside the region of interest and subtracted from the measured intensity.

### Co-immunoprecipitation in HEK293 cells

HEK293 cells (CRL-1573, American Type Culture Collection) were cultured in DMEM with 10% fetal calf serum, 1% penicillin/streptomycin and 1% non-essential amino acids (NEAA) at 37°C and 5% CO_2_. For co-immunoprecipitation, 5×10^6^ HEK293 cells were seeded in T-75 cell culture flasks. The next day, cells were co-transfected with 2 µg of Flag-SF3B1-pcDNA3.1 [a gift from Manoj Pillai (Addgene plasmid #82576; RRID:Addgene_82576) (Kesarwani et al., 2017)] in combination with HA-FBRSL1-I1- pcDNA3.1 or HA-FBRSL1-I3.1- pcDNA3.1 (Ufartes et al., 2020) using jetPRIME *in vitro* DNA & siRNA transfection reagent (Polyplus-transfection) according to the manufacturer's instructions. After 24 h of incubation, cells were detached with trypsin and protein lysis was performed using modified RIPA buffer (1% NP-40, 0.25% sodium deoxycholate, 150 mM NaCl, 1 mM EDTA and 1×protease inhibitor in distilled H_2_O).

For co-immunoprecipitation, 400 µg of the protein lysate diluted in IMAC buffer (20 mM HEPES, 100 mM KCl, 5 mM EGTA, 5 mM MgCl_2_, 1% Triton X-100, 5 mM PMFS, 1 mM DTT and 1× protease inhibitor in distilled H_2_O) was incubated with 5 µl of the monoclonal anti-Flag M2 antibody (F3165, Sigma Aldrich) on a rotator for 3 h at 4°C. Immunoprecipitation was performed by adding 20 µl of protein G magnetic beads (New England Biolabs) to the lysate, followed by an incubation for 2 h at 4°C on a rotator. Prior to washing the beads six times with IP buffer, 39 µl of the supernatant was collected for subsequent western blot analysis. After washing with IP buffer (50 mM Tris, 5 mM MgCl_2_, 150 mM NaCl, 1% Triton X-100 in distilled H_2_O), the beads were washed once with distilled H_2_O. The beads were resuspended in 39 µl distilled H_2_O with 1×NuPAGE LDS Sample Buffer (Thermo Fisher Scientific) and 100 mM DTT, and incubated for 10 min at 70°C. The probes were analyzed by western blotting using the following antibodies: monoclonal anti-HA (C29F4, Cell Signaling, 1:2000), monoclonal anti-Flag M2 (F3165, Sigma Aldrich, 1:1000), anti-rabbit IgG-HRP (A8275, Sigma Aldrich, 1:10,000) and anti-mouse IgG-HRP (A3562, Sigma Aldrich, 1:10,000). Each experiment was conducted with three biological replicates.

### Proximity ligation assay in HeLa cells

HeLa cells (CRM-CCL-2, American Type Culture Collection) were cultured in DMEM with 10% fetal calf serum, 1% penicillin/streptomycin and 1% non-essential amino acids (NEAA) at 37°C and 5% CO_2_. *In situ* Duolink Proximity Ligation Assay (PLA) was performed as previously described (Kastens et al., 2025). HeLa cells were co-transfected with 0.25 µg of Flag-SF3B1-pcDNA3.1 (Kesarwani et al., 2017) in combination with either 0.25 µg of HA-FBRSL1-I1-pcDNA3.1 or HA-FBRSL1-I3.1-pcDNA3.1 (Ufartes et al., 2020). Co-transfections of these plasmids with empty vectors (Flag-pcDNA3.1 and HA-pcDNA3.1) served as negative controls. The following antibodies were used: monoclonal anti-Flag M2 monoclonal anti-HA (C29F4, Cell Signaling, 1:8000) and monoclonal anti-HA (F3165, Sigma Aldrich, 1:500). Duolink PLA was carried out using the *in situ* anti-mouse (minus) and anti-rabbit (plus) PLA probes (Sigma Aldrich), following the manufacturer's instructions. The actin cytoskeleton was counterstained with phalloidin-FITC (P5282, Sigma Aldrich, 1:500), and the cells were mounted using Fluoroshield mounting medium (Sigma Aldrich) containing DAPI to visualize DNA. Immunofluorescence analysis was performed using the Olympus FV1000 confocal laser scanning microscope (EVIDENT) and the FV10-ASW viewer software 4.2 (EVIDENT). Fiji was used for image editing. Each PLA experiment was conducted with two biological replicates.

### Yeast two-hybrid assay

Yeast two-hybrid (Y2H) experiments were performed using the Matchmaker Gold Yeast Two-Hybrid System (Takara). In brief, *Saccharomyces cerevisiae* Y2HGold (Takara) was cultured in 300 ml YPDA medium (Takara) at 37°C until an optical density at 600 nm (OD_600_) of ∼0.5 was reached. Cells were collected by centrifugation at 2500× ***g*** for 5 min, washed twice with distilled H_2_O, and resuspended in 1.5 ml of Tris-EDTA/lithium acetate (LiAc) solution (10 mM Tris, 1 mM EDTA and 100 mM LiAc). The carrier DNA was denatured for 10 min at 95°C prior to transformation. The transformation mixture containing 1 µg of prey and bait expression plasmids plus 10 µl of denatured carrier DNA was added to 100 µl of the cell suspension. Subsequently, 600 µl of polyethylene glycol (PEG)/LiAc solution (10 mM Tris, 1 mM EDTA, 100 mM LiAc and 0.4 g/ml PEG4000) were added, and the mixture was thoroughly mixed by vortexing. The co-transformation of plasmids was carried out by heat shock for 15 min at 42°C. After cooling on ice for 2 min, transformed cells were centrifuged for 1 min at 16,200 ***g***. The supernatant was completely removed, and the cell pellet was resuspended in 150 µl of distilled H_2_O. The cell suspension was plated dropwise onto −leucine/−tryptophan (−LT) and −leucine/−tryptophan/−histidine/−adenine (−LTHA) selective plates. After incubation for several days at 37°C, the plates were imaged using the AZURE c300 chemiluminescent western blot imager with the cSeries Capture Software (Azure Biosystems). Each Y2H experiment was conducted with three biological replicates.

Bait-expressing pGBKT7 vectors with the following inserts were used: human full-length SF3B1 (SF3B1-FL), HEAT domain of human SF3B1 (amino acids 501-1304; SF3B1-HEAT), C-terminal region of human SF3B1 (amino acids 1100-1304; SF3B1-C) and human KDM6A (NM_001291416.2). All SF3B1 inserts contained codon-optimized open reading frames corresponding to NCBI transcript NM_012433.4. Prey-expressing pGADT7 vectors containing the following inserts were used: human FBRSL1 isoform 1 (FBRSL1-I1; NCBI:NM_001142641.2), human FBRSL1 isoform 3.1 (FBRSL1-I3.1; NCBI:NM_001382741.1) and KMD6A-interacting region of KMT2D (amino acids 4294-4879; KMT2D-pt.8; NM_003482.4). Co-transformation of KMD6A with KMT2D-pt.8 served as a positive control (Ufartes et al., 2020). Auto-activation of bait and prey proteins was tested by co-transforming the constructs with the corresponding empty vector.

## Supplementary Material

10.1242/dmm.052472_sup1 Supplementary information
