# Supplementary material for: Fbrsl1 is required for cranial neural crest development and reflects a conserved function of the human disease-associated protein
Source: Dis Model Mech. 2025 Nov 24;18(11):dmm052472. doi: 10.1242/dmm.052472 (PMC12690526; doi:10.1242/dmm.052472)
Supplement: Supplementary information [file dmm-18-052472-s1.pdf]

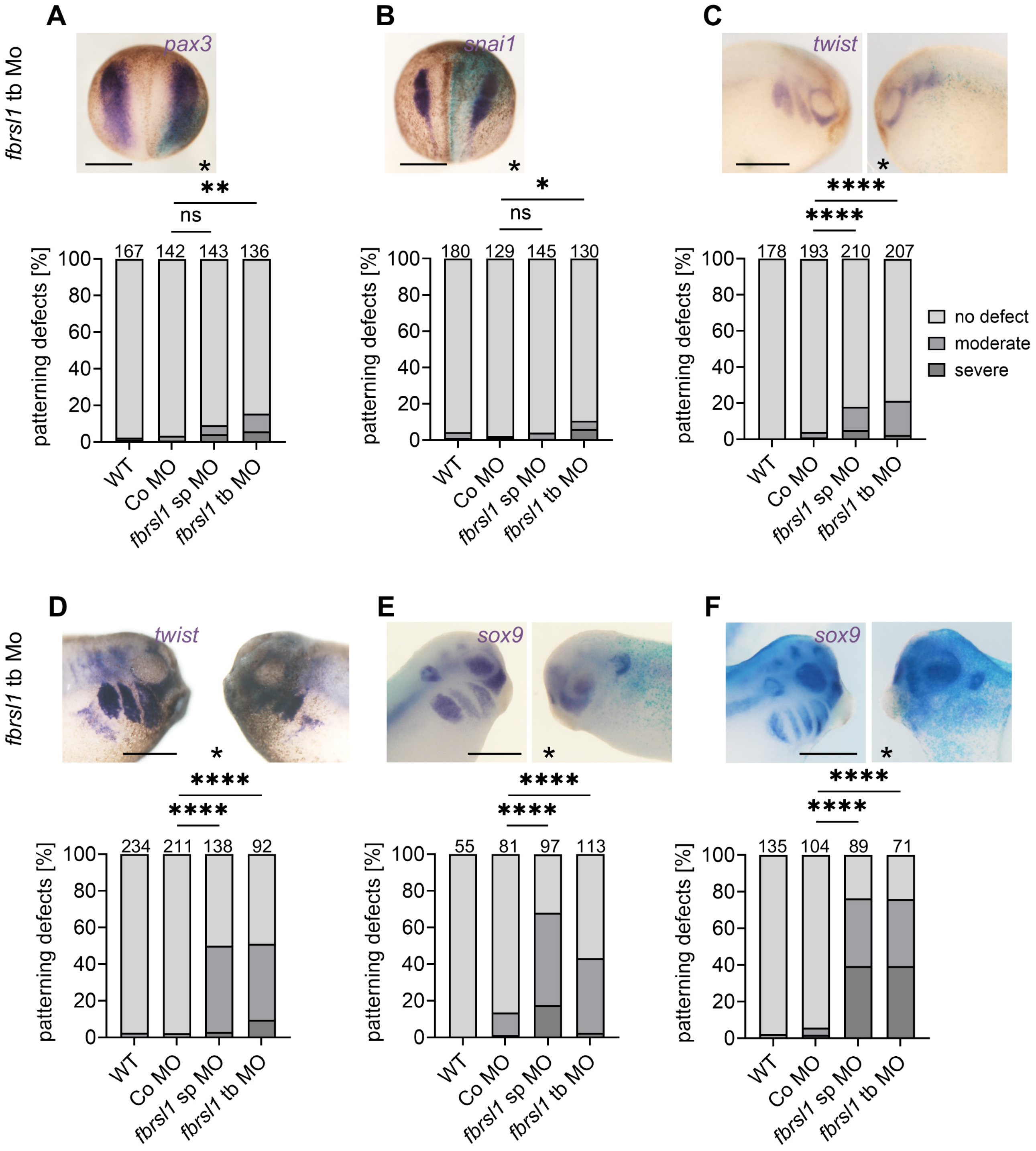

Disease Models & Mechanisms • Supplementary information

Fig. S1. Fbrsl1 is not required for NC induction, specification or early migration, but later stages of NC development. Embryos were injected with 15 ng Co MO, 10 ng *fbrsl1* sp MO or 15 ng *fbrsl1* tb MO and 80 to 100 pg *lacZ* mRNA at the 2-cell stage and analyzed by whole mount *in situ* hybridization using NC markers as indicated. * marks the injected side, which is also visible by the blue LacZ staining. This figure is an extension of Fig. 1. Here only embryos that were injected with the translation blocking *fbrsl1* MO (*fbrsl1* tb MO) are shown. The graphs are identical to Fig. 1 with the addition of the data for the *fbrsl1* tb MO injected embryos. Scale bars: 500 µm. **A** pax3 expression at stage 14**. B** *snai1* expression at stage 16. **C** *twist* expression at stage 23. **D,E** *twist* and *sox9* expression at stage 28. **F** *sox9* expression at stage 33. Data in the graph are presented as percentage of the total, based on at least three independent experiments. The number of analyzed embryos are indicated; ns: not significant, **P*≤ 0.05, ***P*≤ 0.01, *****P*≤ 0.0001 (Two-sided Fisher's exact test).

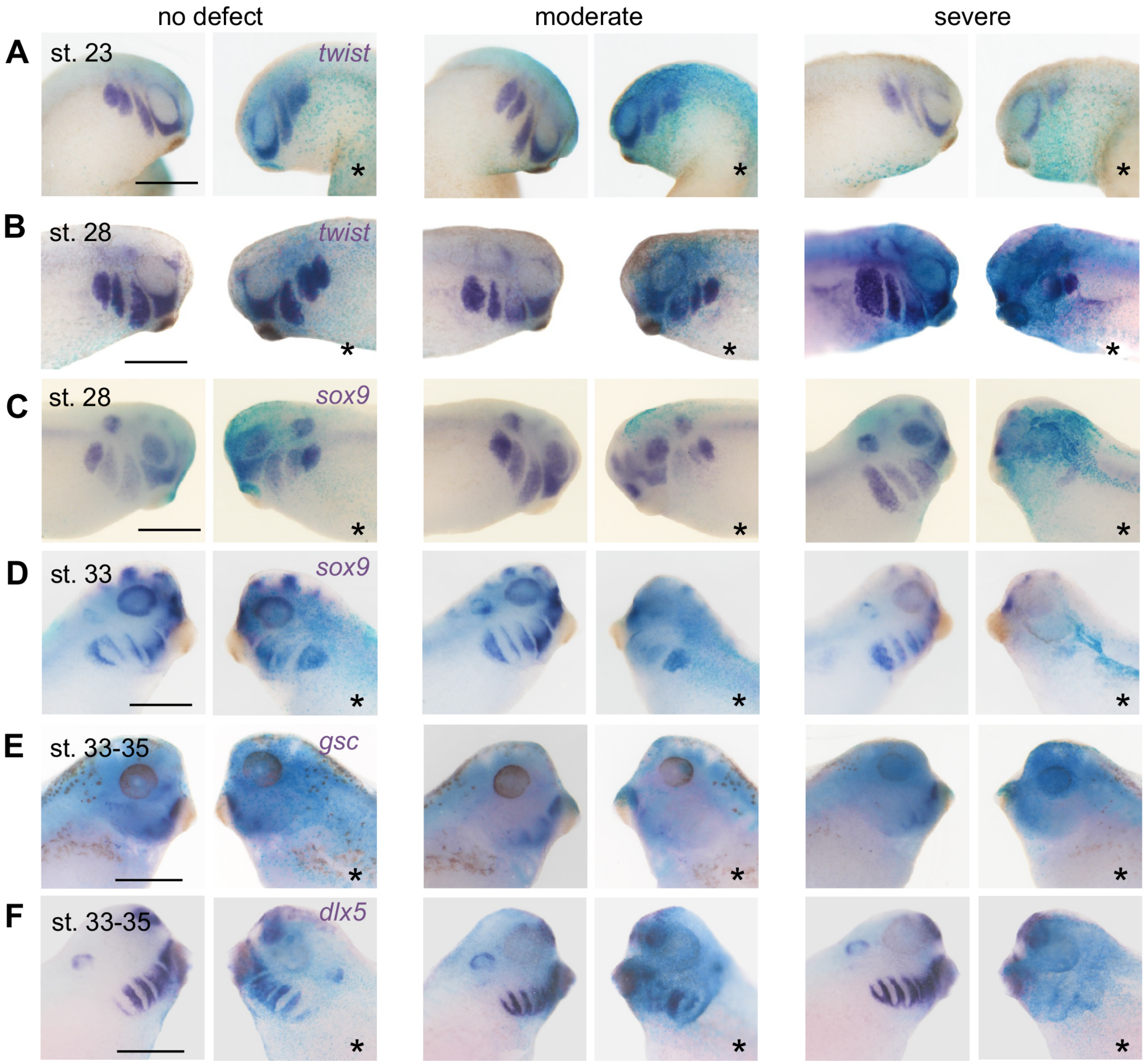

Fig. S2. Categorization of *fbrsl1* morphant phenotypes by *in situ* hybridizations. The panel illustrates how moderate and severe *fbrsl1* morphant phenotypes differ from embryos without detectable defects. *In situ* hybridization was performed at the indicated stages using the following markers: **A,B** *twist*, **C,D** *sox9*, **E** *gsc,* and **F** *dlx5*. Scale bars: 500 µm. 'Moderate' images in E and 'severe' images in F are also shown in Fig. 4.

Disease Models & Mechanisms • Supplementary information

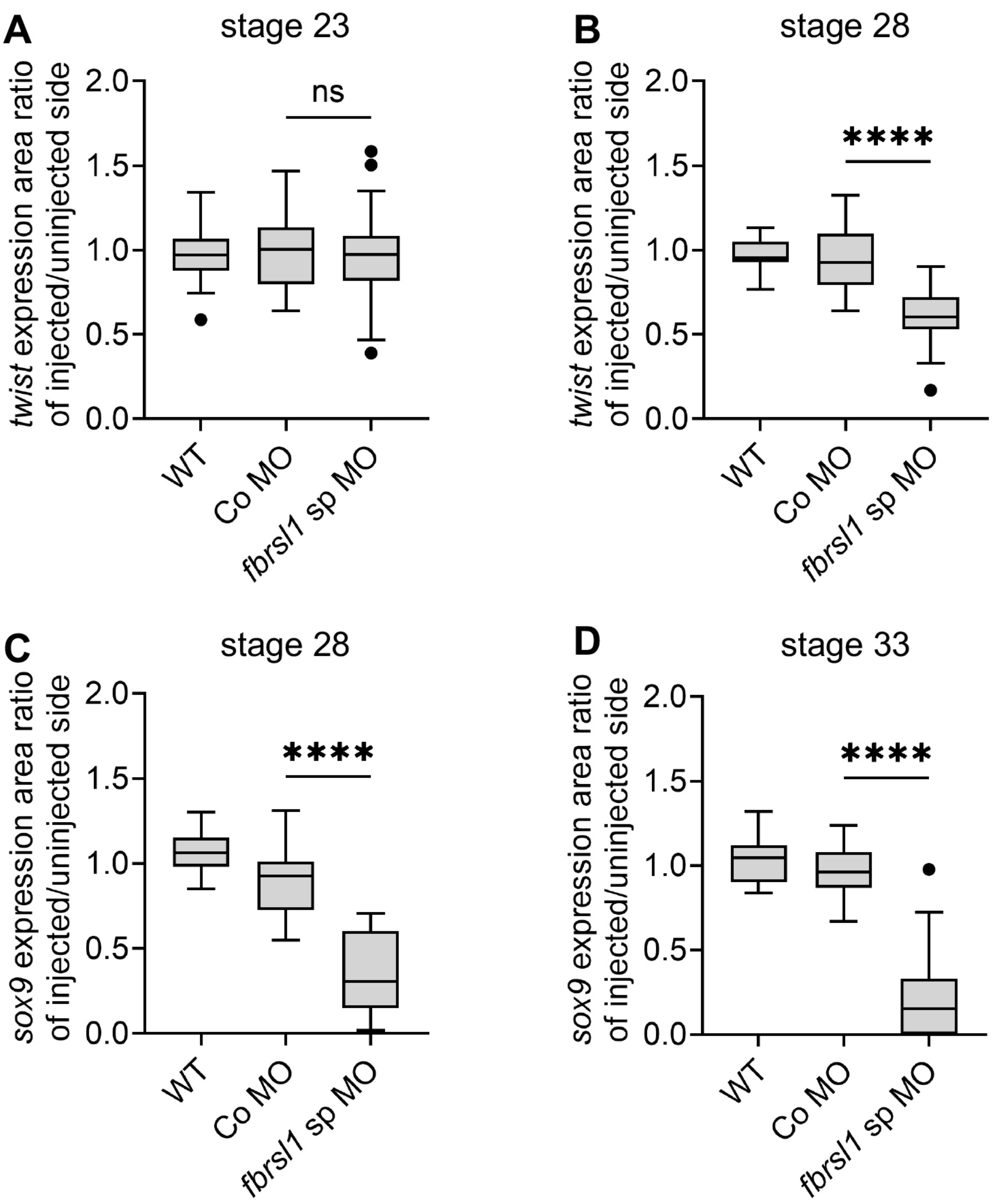

Fig. S3. Quantitative analysis of *twist* and *sox9* expression areas of *fbrsl1* morphant embryos. Graphs show the ratio of the expression area between the injected and the uninjeced side of 20 randomly selected embryos per condition. ns= not significant, ****$P \leq 0.0001$ (one-way ANOVA with Tukey's multiple comparison test, boxplots show median (horizontal line); the boxes extend from the 25th to the 75th percentile, with Tukey's whiskers set at 1.5 times the IQR, dots represent outliers). **A** *twist* expression at stage 23. **B** *twist* expression at stage 28. **C** *sox9* expression at stage 28. **D** *sox9* expression at stage 33.

Disease Models & Mechanisms • Supplementary information

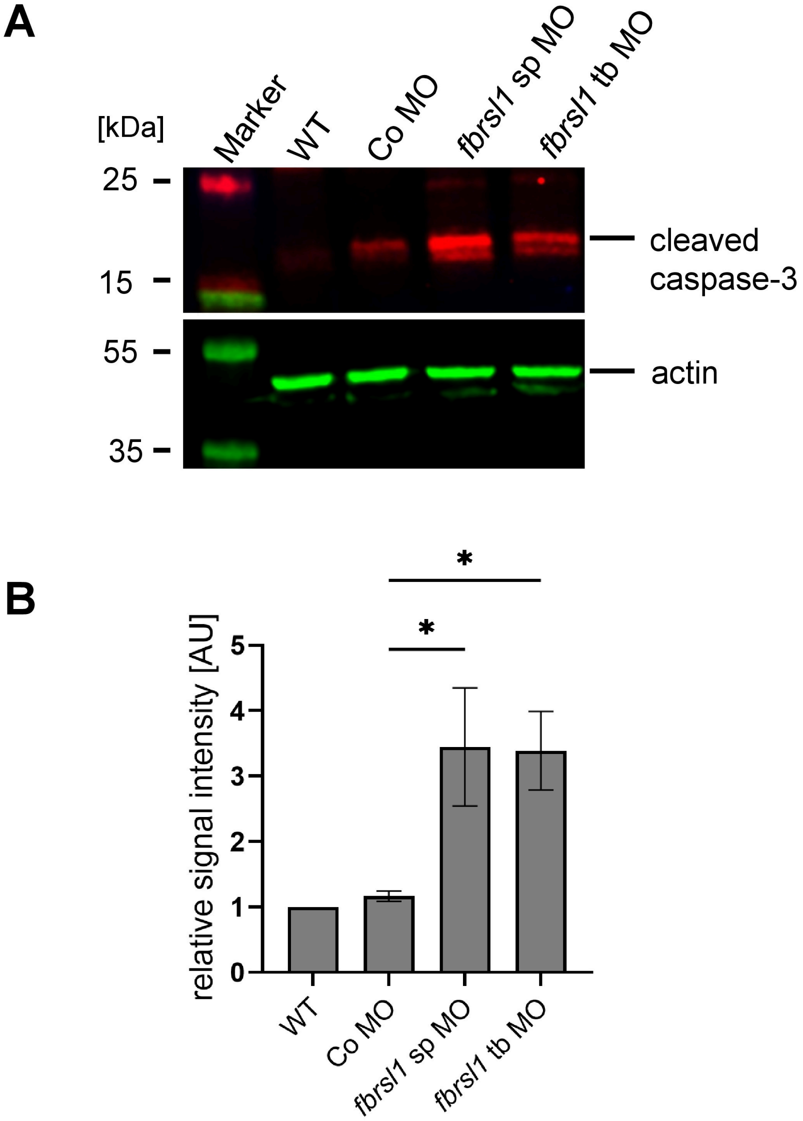

Fig. S4. Cleaved caspase-3 protein levels increase in *fbrsl1* morphant tadpole embryos. *Xenopus* embryos were injected at the one-cell stage with 20 ng of the respective morpholinos and 0.25 µg/µl fluorescein-dextran (Invitrogen) for lineage tracing. **A** The levels of cleaved caspase-3 (at 17 and 19 kDa) were analyzed by western blot at stage 33. A representative western blot is shown. Molecular masses [kDa] are indicated. **B** The ratio of signal intensity levels of cleaved caspase-3 to actin was normalized to WT cleaved caspase-3 levels and plotted. The graph summarizes three independent experiments. Data are mean ± s.e.m; **P*≤ 0.05 (one-way ANOVA with Tukey's multiple comparison test).

Disease Models & Mechanisms • Supplementary information

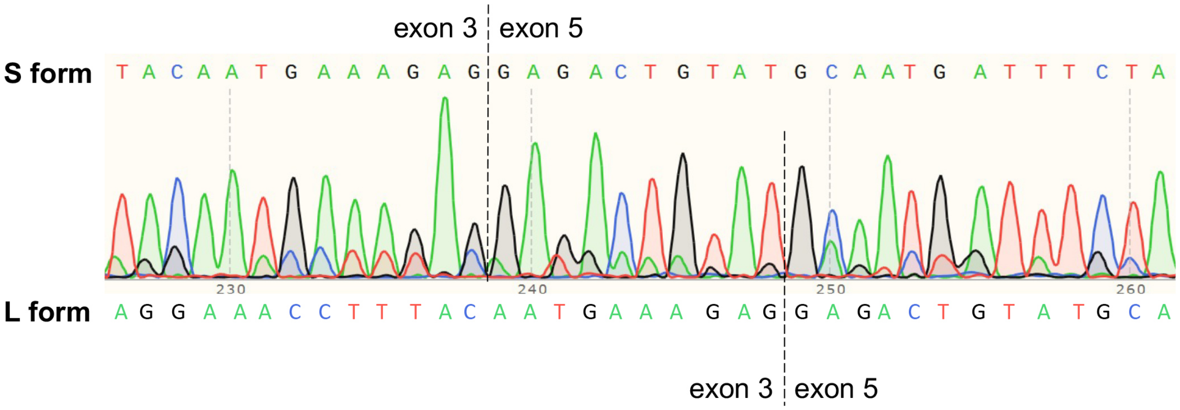

Fig. S5. *Mdm2* exon 4 skipping. *Mdm2* exon skipping was analyzed from cDNA of stage 33 embryos injected with 20 ng Co MO or *fbrsl1* sp MO at one-cell stage. *Mdm2* expression was analyzed at stage 33 by RT-PCR. A smaller *mdm2* band was detected by using primers spanning exon 1 to exon 5 (Fig. 8A) and sequenced. This confirmed *mdm2* exon skipping, as exon 5 directly follows exon 3 in the sequence. The tetraploid *Xenopus laevis* expresses both the S and L forms of *mdm2*, whereby the L form is longer, so that the exon boundaries do not align precisely between the two isoforms.

Disease Models & Mechanisms • Supplementary information

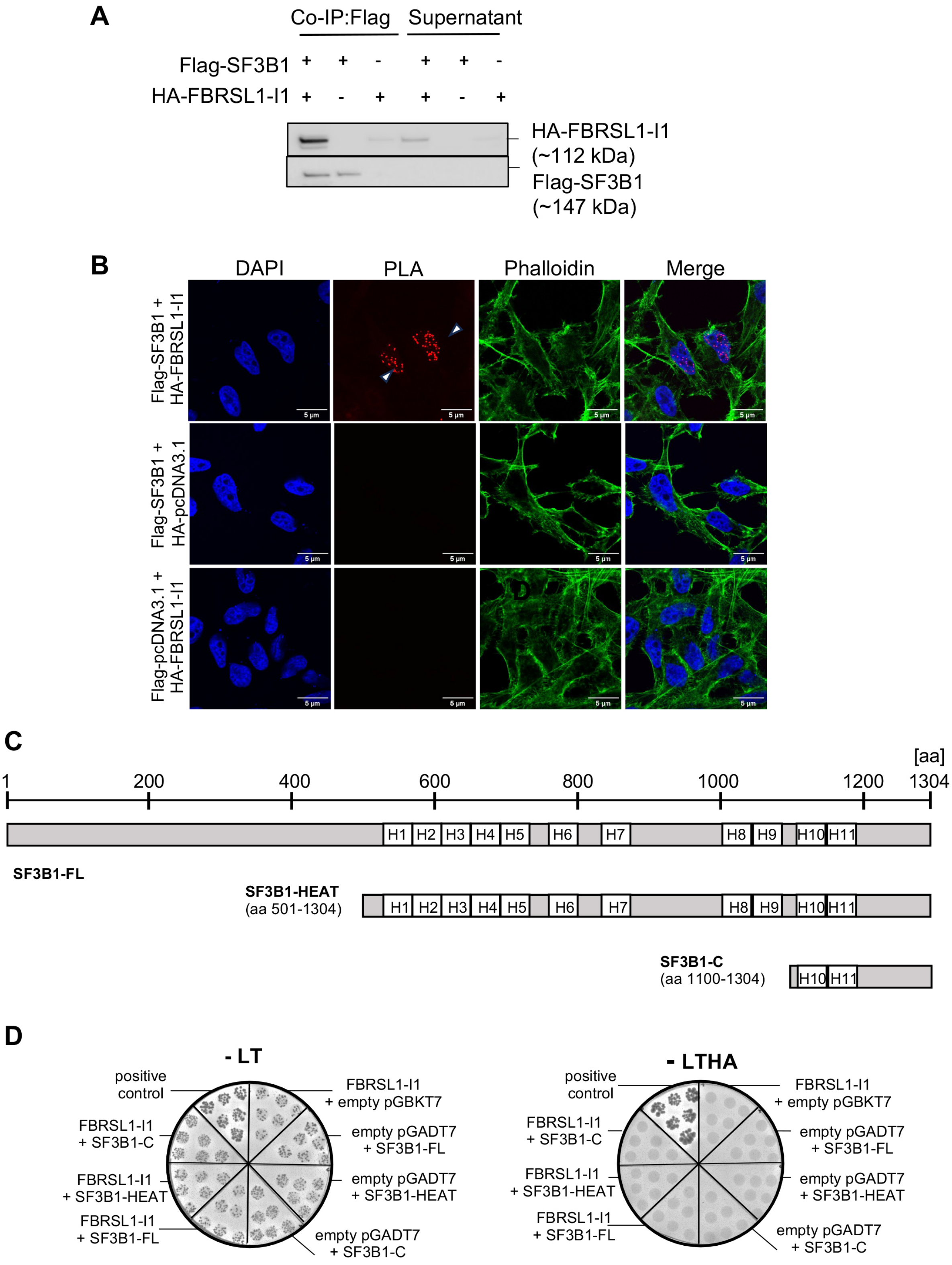

Disease Models & Mechanisms • Supplementary information

Fig. S6. FBRSL1-I1 interacts with the splicing factor SF3B1 in co-immunoprecipitation and PLA assays, but no interaction was observed in yeast two-hybrid (Y2H) assays. A HEK293 cells were co-transfected with Flag-SF3B1 and HA-FBRSL1-I1. Co-immunoprecipitation was performed using an anti-Flag antibody, and precipitated proteins were detected by western blotting; Co IP, Co-immunoprecipitation. **B** Proximity ligation assay (PLA) showing the localization of the FBRSL1-SF3B1 association. Positive PLA signals (indicated by white arrows) were observed after co-transfection of Flag-SF3B1 with HA-FBRSL1-I1, indicating an interaction in the nucleus. Co-transfection with empty vectors (Flag-pcDNA3.1 and HA-pcDNA3.1) served as negative controls. DNA was counterstained with DAPI, and the actin cytoskeleton was visualized using phalloidin. The images for Flag-SF3B1 HA-pcDNA3.1 are also shown in Fig. 8. **C** Schematic illustration of the distinct SF3B1 constructs used for the yeast two-hybrid assays. **D** No direct interaction of FBRSL1-I1 with SF3B1 is seen in Y2H experiments irrespective of the SF3B1 construct used. Co-transformation of KMD6A with KMT2D-pt.8 served as a positive control.

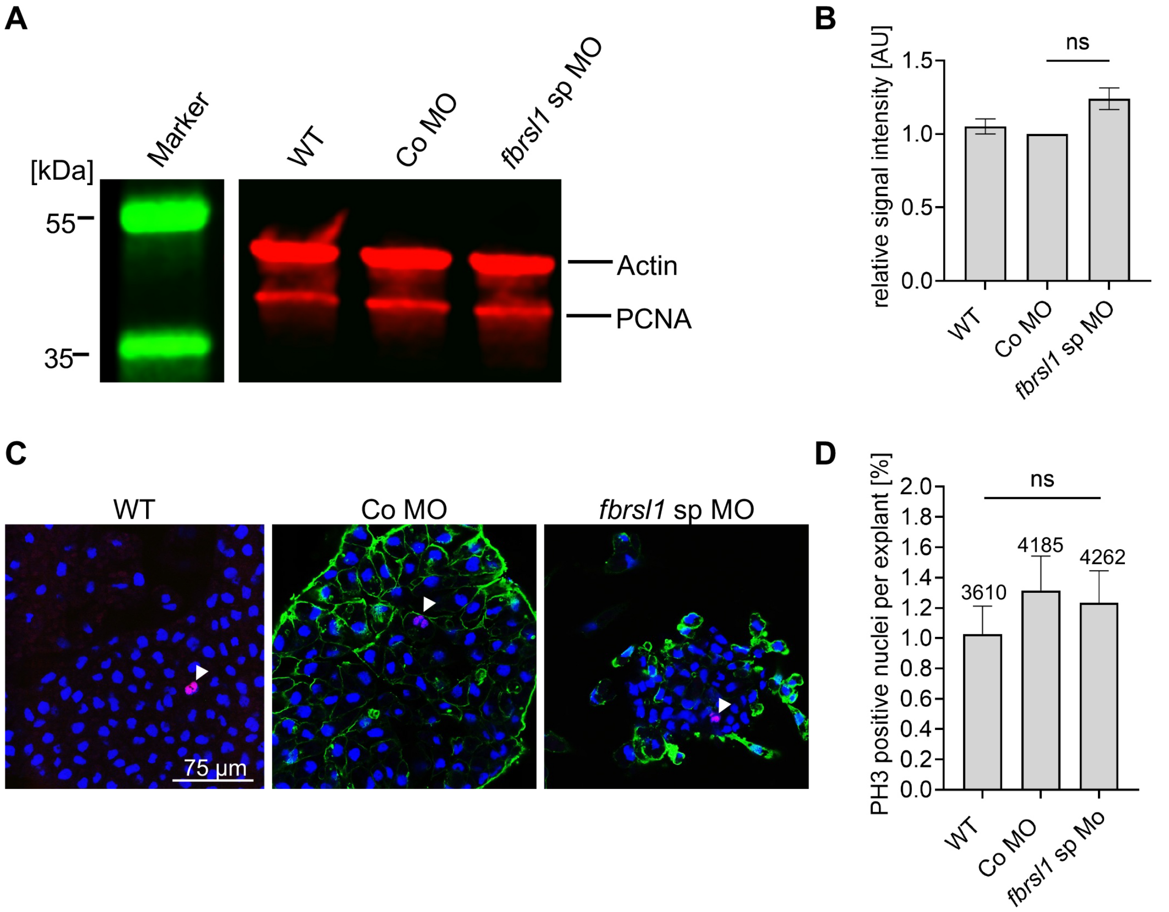

Fig. S7. Fbrsl1 loss of function does not affect proliferation markers in whole embryos or NC explants. **A,B** PCNA expression in whole embryos. Embryos were injected with 20 ng of the respective morpholinos and 0.25 µg/µl fluorescein-dextran for lineage tracing. **A** The expression of PCNA (36 kDa) was analyzed by western blot at stage 33. A representative western blot is shown. Molecular masses [kDa] are indicated. **B** The PCNA-to-actin signal intensity ratio for all samples was normalized to that of CoMO-injected embryos. The graphs summarize three independent experiments, data are mean ± s.e.m; ns, not significant (Kruskal–Wallis test). **C** PH3 expression in NC explants. Embryos were injected with 15 ng Co MO or 10 ng *fbrsl1* sp MO in combination with 100 pg *mbGFP.* NC cells were explanted at premigratory stages and fixed 4 to 6 hours after dissection. The NC cells were analyzed by immunostaining for GFP and PH3. **D** Quantification of PH3-positive nuclei per explant of three independent experiments. Data are mean ± s.e.m.; ns, not significant (Kruskal–Wallis test).

Disease Models & Mechanisms • Supplementary information

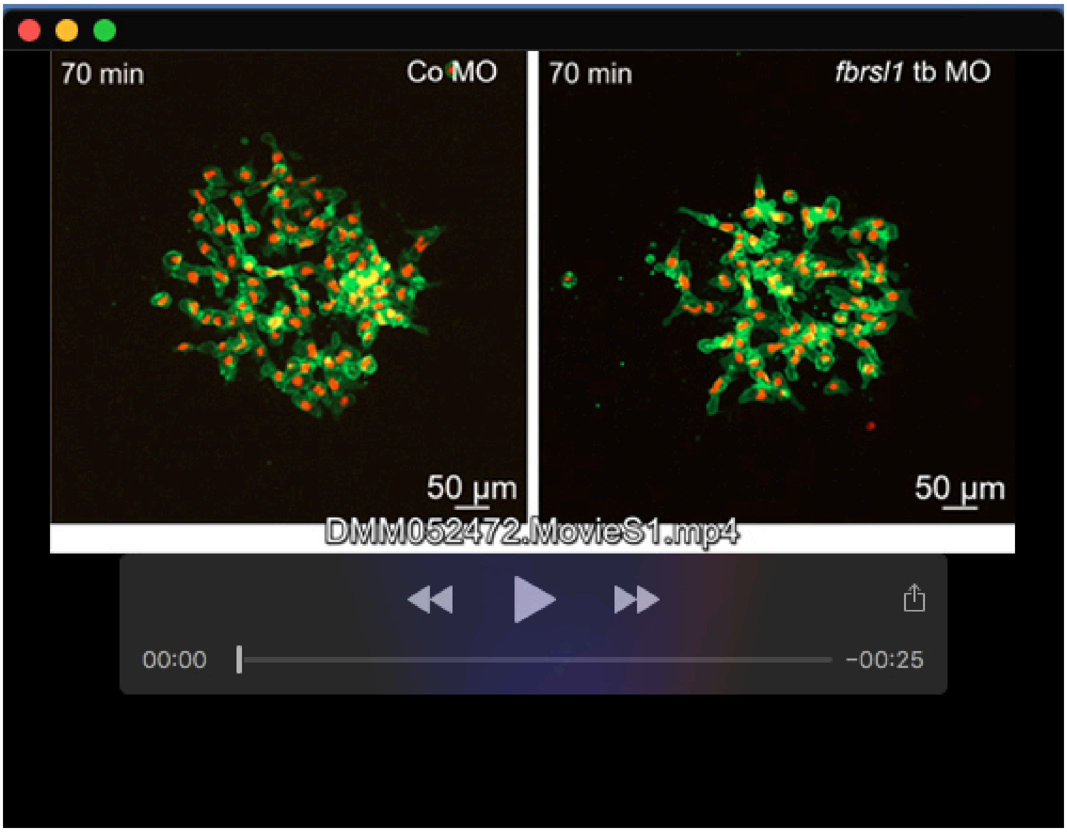

Movie1. f*brsl1* MO-injected NC cells die earlier than Co MO-injected cells.

Embryos were injected with 15 ng Co MO or with 15 ng *fbrsl1* tb MO in combination with 150 pg *mbGFP* RNA and 250 pg *H2B mCherry* mRNA. NC cells were explanted at stage 18, cultivated for 2-3 h and imaged for 5 h using spinning disk microscopy.
